# Homocysteine potentiates amyloid β‐induced death receptor 4‐ and 5‐mediated cerebral endothelial cell apoptosis, blood brain barrier dysfunction and angiogenic impairment

**DOI:** 10.1111/acel.14106

**Published:** 2024-02-15

**Authors:** Ashley Carey, Rebecca Parodi‐Rullan, Rafael Vazquez‐Torres, Elisa Canepa, Silvia Fossati

**Affiliations:** ^1^ Department of Neural Sciences, Alzheimer's Center at Temple Temple University Lewis Katz School of Medicine Philadelphia Pennsylvania USA

**Keywords:** Alzheimer's disease, amyloid beta, angiogenesis, apoptosis, blood brain barrier, cardiovascular risk, cerebral amyloid angiopathy, cerebrovascular dysfunction, endothelial cells, hyperhomocysteinemia

## Abstract

Cerebrovascular dysfunction has been implicated as a major contributor to Alzheimer's Disease (AD) pathology, with cerebral endothelial cell (cEC) stress promoting ischemia, cerebral‐blood flow impairments and blood–brain barrier (BBB) permeability. Recent evidence suggests that cardiovascular (CV)/cerebrovascular risk factors, including hyperhomocysteinemia (Hhcy), exacerbate AD pathology and risk. Yet, the underlying molecular mechanisms for this interaction remain unclear. Our lab has demonstrated that amyloid beta 40 (Aβ40) species, and particularly Aβ40‐E22Q (AβQ22; vasculotropic Dutch mutant), promote death receptor 4 and 5 (DR4/DR5)‐mediated apoptosis in human cECs, barrier permeability, and angiogenic impairment. Previous studies show that Hhcy also induces EC dysfunction, but it remains unknown whether Aβ and homocysteine function through common molecular mechanisms. We tested the hypotheses that Hhcy exacerbates Aβ‐induced cEC DR4/5‐mediated apoptosis, barrier dysfunction, and angiogenesis defects. This study was the first to demonstrate that Hhcy specifically potentiates AβQ22‐mediated activation of the DR4/5‐mediated extrinsic apoptotic pathway in cECs, including DR4/5 expression, caspase 8/9/3 activation, cytochrome‐c release and DNA fragmentation. Additionally, we revealed that Hhcy intensifies the deregulation of the same cEC junction proteins mediated by Aβ, precipitating BBB permeability. Furthermore, Hhcy and AβQ22, impairing VEGF‐A/VEGFR2 signaling and VEGFR2 endosomal trafficking, additively decrease cEC angiogenic capabilities. Overall, these results show that the presence of the CV risk factor Hhcy exacerbates Aβ‐induced cEC apoptosis, barrier dysfunction, and angiogenic impairment. This study reveals specific mechanisms through which amyloidosis and Hhcy jointly operate to produce brain EC dysfunction and death, highlighting new potential molecular targets against vascular pathology in comorbid AD/CAA and Hhcy conditions.

## Graphical abstract

Pictorial representation of the study's results that Hhcy potentiates cerebral endothelial Aβ‐induced DR‐mediated apoptosis, barrier dysfunction, and angiogenesis impairment. High Hcy levels within the cerebral capillary lumen and brain parenchyma and Aβ accumulation in the brain parenchyma and vessel walls potentiate the activation of the DR4/5‐mediated apoptotic pathway, leading to cerebral EC death. Additionally, the combined presence of Hcy and Aβ exacerbate the loss of cerebral EC barrier integrity, leading to BBB permeability. Cerebral EC death and BBB permeability would require activation of angiogenesis to restore homeostasis to the cerebral environment and maintain proper cerebral perfusion, but combined presence of Hcy and Aβ also exacerbate angiogenic deficits. Thus, Hhcy exacerbates Aβ‐mediated cerebral EC dysfunction and potentiates the loss of proper vascular repair mechanisms, leading to a vicious cycle that increases AD pathology.

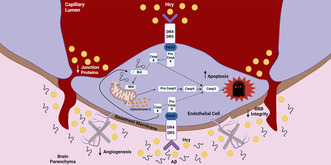

**Abbreviations**
AD: alzheimer's disease
ARIA: amyloid‐related imaging abnormalities
Aβ40: Amyloid‐beta 40
Aβ40‐E22Q: AβQ22
BBB: blood brain barrier
CAA: cerebral amyloid angiopathy
CBF: cerebral blood flow
CV: cardiovascular
CytC: cytochrome c
DR4/DR5: death receptor 4/5
EC: endothelial cell
EEA1: early endosomal antigen 1
HCECs: primary human cerebral endothelial cells
HCMECs/D3: human cerebral microvascular endothelial cells/d3
Hcy: homocysteine
Hhcy: hyperhomocysteinemia
MPTP: mitochondrial permeability transition pore
TEER: trans‐endothelial electrical resistance
TJ: tight junction
TRAIL: TNF‐related apoptosis‐inducing ligand
VEGF‐A: vascular endothelial growth factor A
VEGFR2: vascular endothelial growth factor receptor 2
WT: wild‐type
ZO1: zona occludin 1

## 1 INTRODUCTION

Alzheimer's Disease (AD), the most common age‐associated dementia, is the 6th leading cause of US deaths, currently affecting more than 5.8 million individuals with case numbers exponentially rising (Alzheimer's disease facts and figures, 2020). Pathologically, AD is defined by amyloid beta (Aβ) plaque deposition and neurofibrillary tangles of hyperphosphorylated tau, neuroinflammation, cerebrovascular dysfunction and neuronal death, resulting in cognitive decline (DeTure & Dickson, 2019). Cerebral Amyloid Angiopathy (CAA), a condition involving Aβ deposition (particularly Aβ40 species) around and within the cerebral vasculature, is evident in 85%–95% of AD patients and is also common in the aging population (Cortes‐Canteli & Iadecola, 2020; DeTure & Dickson, 2019; Fossati et al., 2010). CAA pathology contributes to focal ischemia, cerebral blood flow (CBF) and blood brain barrier (BBB) impairments, as well as cerebral hemorrhages (DeTure & Dickson, 2019). Aβ40 is the main component of CAA deposits, and the Aβ40‐E22Q (AβQ22) Dutch familial mutant, containing a mutation of glutamic acid to glutamine on amino acid 22—which accelerates the peptide oligomerization–is highly associated with early onset CAA, encompassing hemorrhagic strokes and dementia in hereditary cerebral hemorrhage with amyloidosis‐Dutch type (HCHWA‐D) (Fossati et al., 2010; Levy et al., 1990). In patients with the Dutch mutation, the effects of CAA are accelerated and exacerbated, with disease onset happening already in the 3rd or 4th decade of life. Hence, the AβQ22 Dutch familial mutant, more aggregation prone and aggressive than Aβ40‐WT (Fossati et al., 2010), constitutes a useful tool to study the effect of toxic Aβ aggregates on the cerebral vasculature in experimental conditions.

Recent evidence suggests that cerebrovascular disturbances may precede Aβ‐mediated pathology in AD (Korte et al., 2020; Nortley et al., 2019), shifting the focus of the dementia field to understanding the pathological contribution of cardiovascular (CV) factors to AD onset and progression (Iturria‐Medina et al., 2016; Love & Miners, 2016). Indeed, it has been demonstrated that CV risk factors can promote AD pathology and increase individuals' AD risk (Carey & Fossati, 2023; Love & Miners, 2016).

Multiple CV risk factors have been shown to cause endothelial cell (EC) dysfunction leading to cerebral hypoperfusion, which is an early contributor to AD pathology (Di Marco et al., 2015) and BBB abnormalities (Wong et al., 2019). Hyperhomocysteinemia (Hhcy), a disorder involving excess plasma homocysteine (Hcy) levels due to vitamin deficiencies and Hcy metabolism disruption, is prevalent in 5%–7% of the US population, and this prevalence increases with age (Son & Lewis, 2023). Hhcy is recognized as both a CV risk factor and a risk factor for AD and vascular dementia (Carey & Fossati, 2023; Kamat et al., 2015; Tinelli et al., 2019). It is also known to promote oxidative stress, vascular inflammation, and EC dysfunction (Balint et al., 2020; Lai & Kan, 2015; Tinelli et al., 2019). Hhcy and amyloidosis both have detrimental effects on ECs function and health and are both thought to cause cerebrovascular damage. Despite the compelling data in favor of Hcy as a modifiable risk factor, the debate regarding the significance of Hcy mediated health effects, and if these are causal for cerebrovascular dysfunction is still ongoing. Moreover, whether the molecular mechanisms through which endothelial damage is caused are overlapping in CAA and Hhcy remains mostly unknown. Identifying specific pathways activated by both Hhcy and Aβ to promote cerebrovascular pathology and understanding whether there is an additive or synergistic activation of the same molecular mechanisms in ECs will be vital for identifying new therapies for these frequently comorbid conditions, which are increasingly common in aging individuals.

Cerebral ECs are connected by tight junction (TJ) proteins (such as occludin and claudin5), as well as anchor proteins [such as zona occludens‐1 (ZO1)] and cadherin proteins (VE cadherin) (Yu, Ji, & Shao, 2020). Not only are brain ECs vital for the formation of a physical barrier to protect the cerebral environment, but they contribute to the transport in and out of the brain, by regulating influx and efflux, and to the immunological barrier, by preventing immune cell infiltration and neuroinflammation (Yu et al., 2020). BBB dysfunction is a prominent feature of AD pathology, involving disruption of TJs and loss of barrier integrity. Increased BBB permeability leads to the leakage of neurotoxic blood proteins and immune cells, promoting neuroinflammation and oxidative stress. As our lab and others have demonstrated, AβQ22 and other Aβ peptides directly contribute to BBB permeability (Cuevas et al., 2019; Hartz et al., 2012; Parodi‐Rullan et al., 2020).

We have also demonstrated that Aβ oligomers activate caspase‐ and mitochondria‐mediated apoptosis in cerebral ECs through their direct binding with the TNF‐related apoptosis‐inducing ligand (TRAIL) death receptors (DRs), DR4, and DR5 (Fossati et al., 2012b). Ligand binding to DR4/5 results in the activation of caspase 8, to initiate the extrinsic apoptotic pathway (Elmore, 2007). Activated caspase 8 cleaves Bid into activated tBid. tBid increases mitochondrial membrane permeability, inducing release of cytochrome c (CytC), which causes caspase 9 activation (Elmore, 2007). Data from our lab and others have shown that Aβ induces caspase 8 activation and CytC release into the cytoplasm in cerebral ECs (Fossati et al., 2012a; Solesio et al., 2018; Xu et al., 2001), eventually resulting in caspase 3 activation and, ultimately apoptotic cell death (Elmore, 2007), and knocking down DR4/5 resulted in 60%–70% protection from Aβ‐induced EC apoptosis (Fossati et al., 2012b). As the kinetics for these affects are aggregation‐dependent, DRs activation occurs more rapidly in AβQ22‐treated ECs, although the same pathways are activated in ECs challenged with Aβ40‐WT, once it forms high molecular weight oligomers or protofibrils (Fossati et al., 2012b).

Cerebral angiogenesis, the process of forming new blood vessels, is vital for maintenance of homeostasis within the cerebral vasculature and in the brain parenchyma (Beck & Plate, 2009). Typically, cerebral angiogenesis is activated during hypoxia and hypoperfusion as a mechanism to maintain proper CBF (Beck & Plate, 2009). Previous research has demonstrated that Aβ40‐WT and AβQ22 are capable of 90–100% inhibition of EC proliferation, disrupting EC response to angiogenic stimuli (Solito et al., 2009). More recently, our lab has demonstrated that AβQ22 has potent inhibitory effects on EC angiogenesis, compared to other Aβ peptides (Parodi‐Rullan et al., 2020). Aβ may reach very high concentrations in CAA deposits around the vessels, impairing neo‐angiogenesis, which is vitally needed as a repair mechanism, especially when brain vessels are injured by cerebrovascular risk factors, such as Hhcy (Parodi‐Rullan et al., 2020).

Hence, in this study, we aim to understand whether high levels of Hcy potentiate the effects of Aβ on DR‐mediated EC death, BBB dysfunction and angiogenic pathways. The goal of this study is to elucidate whether combined exposure of ECs to Aβ and Hcy promotes exacerbated levels of BBB damage by acting on the same molecular and cellular mechanisms. Overall, we hypothesize that Hhcy potentiates Aβ‐mediated TRAIL DR‐dependent EC apoptosis, BBB permeability, and angiogenic impairment through additive mechanisms, accelerating the progression of cerebrovascular pathology and, ultimately, increasing the risk for AD and dementia.

## 2 MATERIALS AND METHODS

### 2.1 Cell culture

Immortalized human cerebral microvascular endothelial cells (HCMEC/D3) were obtained from Babette Weksler (Cornell University) (Fossati et al., 2010). Cells were grown in EBM‐2 (Lonza) and supplemented with growth factors (Hydrocortisone, hFGF‐B, VEGF, R3‐IGF‐1, ascorbic acid, hEGF, and GA‐1000) and 5% FBS and maintained at 37°C in a humidified cell culture incubator under a 5% CO_2_ atmosphere. Cells were visualized and imaged using the EVOS M5000 Imaging System (Thermo Fisher Scientific). For the TJ staining, primary human cerebral endothelial cells (HCECs) (Sciencell) were utilized and maintained under the same conditions as the HCMECs.

### 2.2 AβQ22 peptide

For treatments, we utilized the genetic variant of the wild‐type (WT) Aβ40 peptide containing the E22Q substitution (AβQ22), which is the synthetic homolog of the amyloid subunit present in the vascular deposits in sporadic and familial Dutch‐AD cases. The peptide was synthesized by Peptide 2.0. AβQ22 was dissolved to 1 mM in 1,1,1,3,3,3‐hexafluoro‐2‐propanol (HFIP; Sigma, St. Louis, MI, USA), incubated for 24 h to breakdown pre‐existing β‐sheet structures and lyophilized. Peptides were subsequently dissolved in DMSO to a 10 mM concentration, followed by the addition of deionized water to 1 mM concentration, and further diluted into culture media (EBM‐2 (Lonza) with 1% FBS and no growth factors) to the required concentrations for the different experiments.

### 2.3 Homocysteine

Hcy was weighed and exposed to 0% O_2_ and 5% CO_2_ conditions in a hypoxic chamber (Coy Laboratory Products) located in a 37°C humidified cell culture incubator for 15 min to prevent oxidation before cell treatment. Hcy was prepared fresh for each treatment and dissolved in media to obtain a 100 mM concentration and further dissolved to obtain a 10 mM concentration. The 10 mM Hcy solution was filtered, and additional media was added to obtain the required concentration for the different experiments.

### 2.4 Mouse models

Male and female Tg2576 mice, a widely used model of cerebral amyloidosis expressing the Swedish mutation (K670N/M671L), and age‐matched WT littermates were bred internally. Mice were maintained under controlled conditions (~22°C, and in an inverted 12‐h light–dark cycle, lights on 10 am to 10 pm) with unrestricted access to food and water. The generation of B6; SJL‐Tg (APPSWE) 2576Kha mice (Tg2576) on a B6; SJL Mixed Background was as described (Hsiao et al., 1996). Tg2576 mice develop parenchymal Aβ plaques at 11–13 months and vascular Aβ deposition (CAA) at 10–11 months of age (Robbins et al., 2006). Starting at 5 months, WT and Tg2576 mice were supplemented with a Hhcy‐inducing diet, consisting of high methionine (Hcy precursor; 20 g/kg) and low folate (3.2 mg/kg), vitamin B6 (15 mg/kg), and vitamin B12 (55 ug/kg) (needed to metabolize Hcy). Mice were sacrificed at 13–14 months of age and tissue lysate from the prefrontal cortex was harvested. All experiments and animal protocols were performed according to protocols approved by the Institutional Animal Care and Use Committee of Temple University School of Medicine and conformed to the National Research Council Guide for the Care and Use of Laboratory Animals published by the US National Institutes of Health (2011, eighth edition).

### 2.5 Western blot

Evaluation of DR4 (Invitrogen; 32A242), DR5 (Enzo; ALX‐210‐743‐C200), cFLIP (Cell Signaling; CS563435), BCL2 (abcam; ab196495), Bax (Novus; NBPI‐88682), ICAM (Invitrogen; MA5407), ZO1 (Invitrogen; 61‐7300), Claudin5 (Invitrogen; 35‐2500 4C3C2), phosphorylated Claudin5 (abcam; ab172968), phosphorylated VeCadherin (Invitrogen; 44‐11,446), MMP2 (abcam; ab86607), VEGFR2 phospho Y1175 (abcam; ab194806), and VEGF‐A (Proteintech; 19,003‐1‐AP) was performed using WB analysis after electrophoretic separation on 4%–12% Bolt Bis‐Tris SDS polyacrylamide gels. Evaluation of Bid/tBid (Cell Signaling; CS#2002) and cleaved caspase 3 (Cell Signaling; C#9664) was performed using WB analysis after electrophoretic separation on Novex WedgeWell 4%–20% tris‐glycine SDS polyacrylamide gels. Normalization was performed using anti‐β actin (Millipore; MAB1501) or anti‐GAPDH (Cell signaling; 32,233). Proteins were electrotransferred to nitrocellulose membranes (0.45 μM pore size; Amersham, Cytiva LifeSciences) at 110 V for 70 min, using towbin buffer, containing 20% (v/v) methanol. Membranes were blocked with 5% non‐fat milk in TBST (or 5% BSA in TBST for the phosphorylated proteins) containing 0.1% Tween 20, and subsequently immunoreacted with the respective primary antibodies for each experiment, followed by incubation with the appropriate anti‐rabbit, anti‐mouse, or anti‐goat secondary antibodies (1/20,000; LICOR). Membranes were developed with the LICOR Odyssey CLx Immunoblot Imager and blots were analyzed with the LICOR Image Studio software.

### 2.6 Death receptor 4 and 5 silencing

Silencing of DR4 (TNFRSF10A: siRNA ID#s16764) and DR5 (TNFRSF10B: siRNA ID#s16756) was achieved using Ambion Silencer siRNA according to the manufacturer's recommendations. HCMECs were seeded to obtain 70% confluency in 24 h. Cells were then transfected with Lipofectamine RNAiMAX (Invitrogen) and Ambion Silencer siRNA (Life Technologies) at a final concentration of 10 μM in Opti‐MEM Reduced Serum Medium (Gibco, Life Technologies). 4 h post‐transfection, the cells were supplemented with complete media for 24 h. Following the 24 h, transfection media was removed, and cells were maintained in complete media until the treatment for the experimental endpoints. The efficiency and specificity of silencing DR4 (ThermoFischer; Hs00269492_m1) and DR5 (ThermoFischer; Hs00366272_m1) (72 h post‐transfection) was confirmed by quantitative real‐time polymerase chain reaction (qRT‐PCR) and samples were normalized to cyclophilin‐B (ThermoFischer; Hs00168719_m1). RNA was extracted utilizing the miRNeasy kit (Qiagen). cDNA was obtained using the SuperScript IV VILO (Invitrogen) reverse transcription kit according to the manufacturer's protocol.

### 2.7 Cell death ELISA

Apoptotic cell death was assessed as formation of fragmented nucleosomes using the Cell Death Detection ELISA^Plus^ kit (Roche Applied Science) according to the manufacturer's instructions. Briefly, HCMEC were seeded and, after 24 h, treated with 25 μM AβQ22, 1 mM Hcy, or a combination of both, in EBM‐2 media supplemented with 1% FBS. Extranuclear DNA‐histone complexes were measured with Cell Death Detection ELISA^Plus^ at 405 nm using the SpectraMax i3x Multi‐Mode Microplate Reader (Molecular Devices). Results are expressed as percent change compared with untreated control cells.

### 2.8 Propidium iodide (PI) necrosis staining

Necrosis levels were fluorescently assessed using a PI Nucleic Acid Live Cell Stain (Invitrogen). 10,000 cells/well were seeded in a 96 well plate and, following 24 h, were treated with 25 μM AβQ22, 1 mM Hcy, or a combination of both for 24 h. A stock of 1 mg/mL PI was dissolved in dH_2_o. Following treatment, the media was removed, and the PI solution was added to each well as a 1:200 dilution in cell culture media. Cells were incubated with the dye for 1 h at room temperature in the dark and were imaged with bright field and an RFP filter using an EVOS M5000 imaging system. Four randomized images were taken per well. The percentage of PI positive cells was calculated by counting the total number of cells present in each picture and creating a mask for the fluorescent signal to count the number of cells showing PI fluorescence [% of PI Positive Cells = (# of cells with PI/total number of cells) × 100].

### 2.9 Caspase‐3/7, −8, and − 9 activity assays

Caspase activation was measured by luminescent assays (Caspase‐Glo‐3/7, −8 or − 9, Promega, Madison, WI, USA), in cells treated for 8 h with 50 μM AβQ22, 1 mM Hcy, or a combination of both in EBM‐2/1% FBS. Briefly, 10,000 cells/well were seeded in white wall/bottom 96‐well plates and were treated following 24 h. Caspase‐Glo reagent was added to the cell cultures resulting in cell lysis, followed by caspase cleavage of the substrate and generation of a luminescent signal produced by the luciferase reaction. After 1 h incubation, the signal, proportional to the amount of caspase activity present, was evaluated in a plate‐reading luminometer (SpectraMax i3x Multi‐Mode Microplate Reader; Molecular Devices). To inhibit nonspecific background activity, the proteasome inhibitor MG‐132 was added to the Caspase‐Glo 8 and 9 reagent before the experiment as indicated by the manufacturer. In all cases, results are expressed as percent change compared with untreated control cells.

### 2.10 Cell event fluorescent caspase 3/7 assay

Cleaved/Active Caspase 3/7 expression was assessed using the CellEvent™ Caspase‐3/7 Green Detection Reagent (ThermoFischer Scientific). 10,000 cells/well were seeded in a 96 well plate and were treated with 25 μM AβQ22, 1 mM Hcy, or a combination of both following 24 h. Following the treatment, the media was removed, and the fluorescent Caspase 3/7 Green Detection Reagent was added to each well at a concentration of 5 μM. Cells were incubated with the dye for 1 h at 37°C and were imaged with bright field and a GFP filter using an EVOS M5000 imaging system. Four randomized images were taken per well. The percentage of cleaved caspase 3/7 positive cells was calculated by counting the total number of cells present in each picture and creating a mask for the fluorescent signal to count the number of cells showing active caspase 3/7 fluorescence [% of Caspase 3/7 Positive Cells = (# of cells with cleaved caspase 3/7/total number of cells) × 100].

### 2.11 CytC immunocytochemistry

Release of CytC from the mitochondria into the cytoplasm was assessed via immunocytochemistry. HCMECs were seeded in eight well chamber slides (Millipore) coated with Attachment Factor (Cell systems) and, after 24 h, treated with 25 μM AβQ22, 1 mM Hcy, or a combination of both for 6 h. Following treatment, cells were stained with MitoTracker Red CM‐H_2_XRos (Invitrogen), a dye that enters the mitochondria only if they present a healthy membrane potential, for 30 min at 37°C. Cells were then fixed with 4% PFA (BeanTown Chemical) and permeabilized with 0.2% triton‐x100 in PBS for 10 min at room temperature. After blocking with 3% BSA in PBS for 1 h, cells were stained with CytC alexaflour488 (1:500) (BD Biosciences; 560,263) in 1% BSA in PBS for 1 h. Following the staining, cells were mounted with DAPI (blue) mounting media (SouthernBiotech; 0100–20) and images were taken with a fluorescent inverted microscope (Nikon Eclipse Ti2) using the NIS Elements AR Analysis 5.360.02 software and NIS Elements AR 5.360.02 image capturing software.

### 2.12 CytC ELISA

Release of CytC from the mitochondria into the cytoplasm was assessed by a human CytC Quantikine ELISA kit (Bio‐techne R&D Systems) according to the manufacturer's recommendations. HCMECs were seeded and following 24 h treated with 25 μM AβQ22, 1 mM Hcy, or a combination of both for 6 h. Mitochondrial isolation was conducted in order to obtain mitochondrial and cytoplasmic fractions. Data is represented as the ratio of cytoplasmic CytC/mitochondrial CytC (percent change from control).

### 2.13 ECIS trans‐endothelial electrical resistance (TEER)

Cerebrovascular endothelial barrier formation was assessed using the ECIS Zθ system (Applied Biophysics). All experimental procedures were performed in 8‐well ECIS (8WE10+, Applied Biophysics) 40‐electrodes‐gold plated arrays pre‐treated according to the manufacturer's instructions. A monodisperse solution of HCMECs was seeded and monitored for 48 h until the electrical resistance reached a plateau at a frequency of 4000 Hz, indicative of barrier formation. At this point, the cell monolayers were treated with 5 or 10 μM AβQ22, 1 mM Hcy, or a combination of 5 or 10 μM AβQ22 and 1 mM Hcy in EBM‐2 media containing 1% FBS and followed for 48 h posttreatment. Barrier permeability was assessed as a decrease in barrier resistance at 4000 Hz compared with untreated cells.

### 2.14 ZO1 immunocytochemistry

ZO1 expression and localization was assessed via immunocytochemistry. Primary HCECs were seeded in eight well chamber slides (Millipore) coated with collagen I (0.15 mg/mL) and grown until 100% confluence in complete media. Once confluent, cells were exposed to EBM2 media containing 0.25% FBS and bFGF (1:2000) for 24 h to promote TJ formation. Cells were then treated with 10 μM AβQ22, 1 mM Hcy, or a combination of both for 48 h. Following treatment, cells were fixed with 4% PFA (BeanTown Chemical) for 10 min at room temperature and blocked overnight at 4°C in 20 mg/mL BSA. Cells were then incubated with ZO1 primary antibody (Invitrogen; 61‐7300) for 1.5 h at room temperature (1:100 in 5 mg/mL BSA 0.05% triton‐x100). Subsequently, cells were incubated with alexaflour 568 goat anti‐rabbit secondary antibody (Life Technologies, A11011) for 1 h at room temperature in the dark (1:200 in 5 mg/mL BSA 0.05% triton‐x100). Cells were then stained with Phalloidin alexaflour488 (Invitrogen; A12379) for 30 min at room temperature in the dark (1:100 in 5 mg/mL BSA). Following the staining, cells were mounted with DAPI (blue) mounting media (SouthernBiotech; 0100‐20) and images were taken with a fluorescent inverted microscope (Nikon Eclipse Ti2) and deconvolved with the 64‐bit NIS Elements AR Analysis 5.360.02 analysis software and NIS Elements AR 5.360.02 image capturing software. Area of ZO1 and Phalloidin fluorescence was analyzed with Halo software (Indica Labs).

### 2.15 MMP2 activity assay

The levels of active MMP2 were measured with a MMP2 activity assay (QuickZyme Biosciences) according to the manufacturer's recommendations. HCMECs were seeded (350,000 cells/well in six well plates) and following 24 h treated with 25 μM AβQ22, 1 mM Hcy, or a combination of both for 6 h. Following treatment, cells were lysed, and protein extracts were stored at −80°C until the assay was conducted.

### 2.16 Angiogenesis inhibition assay

Inhibition of angiogenesis was assessed using Millipore's Millicell μ‐Angiogenesis Inhibition Assay according to the manufacturer's recommendations. HCMEC suspensions were seeded in the presence/absence of 1 μM AβQ22, 500 μM Hcy, or a combination of both in a Millicell μ‐Angiogenesis Slide containing ECMatrix Gel Solution. Sulforaphane was used as a positive control of angiogenesis inhibition. In all cases, tube formation was monitored after 4 h by acquiring pictures with an EVOS M5000 imaging system. Capillary branches meeting length criteria were counted from four randomized images for each treatment well. Angiogenesis Progression Score was determined by a scale provided from the manufacture (0 = lowest progression level and 5 = highest progression level).

### 2.17 ECIS wound healing assay

EC wound healing capability was assessed using the ECIS Zθ system (Applied Biophysics). All experimental procedures were performed in 8‐well ECIS (8W1E, Applied Biophysics) gold plated arrays, presenting a single central electrode, pre‐treated according to the manufacturer's instructions. A monodisperse solution of HCMECs was seeded and monitored for 48 h until the electrical resistance reached a plateau at a frequency of 4000 Hz, indicative of barrier formation. At this point, the cell monolayers were treated with 10 μM AβQ22, 1 mM Hcy, or a combination of both in EBM‐2 media containing 1% FBS. Approximately 1 h‐post treatment, a 20 s wound was inflicted to the ECs (60,000 Hz; amplitude 5 V; wound current = 1400uAmps) and wound healing was followed for 48 h post‐injury. Impaired wound healing was defined as decreased barrier resistance following injury compared with untreated cells.

### 2.18 VEGF‐A ELISA

Levels of soluble VEGF‐A were assessed by a Human VEGF‐A Quantikine ELISA kit (Bio‐techne R&D Systems), according to the manufacturer's recommendations. HCMECs were seeded and following 24 h treated with 25 μM AβQ22, 1 mM Hcy, or a combination of both for 24 h. Following treatment, media was collected and stored at −80°C until the ELISA was conducted.

### 2.19 VEGFR2 and EEA1 immunocytochemistry

VEGFR2 expression and localization was assessed via immunocytochemistry with an EEA1 co‐stain. HCMECs were seeded in eight well chamber slides (Millipore) and, after 24 h, treated with 25 μM AβQ22, 1 mM Hcy, or a combination of both for 6 h. Following treatment, cells were fixed with 4% PFA (BeanTown Chemical). Cells were permeabilized with 0.2% triton‐x100 in PBS for 10 min at room temperature. Cells were blocked with 3% BSA in PBS for 1 h and cells were stained with VEGFR2 (Cell Signaling; C#2479; 1:200) and EEA1 (R&D Systems; AF8047; 5ug/mL) primary antibodies in 1% BSA in PBS overnight at 4°C. Cells were then incubated with secondary antibodies (anti‐sheep586 (abcam) and anti‐rabbit488 (Invitrogen); 1:200) in 1% BSA/PBS with 0.3% triton‐x100 for 2 h at room temperature. Following the staining, cells were mounted with DAPI (blue) mounting media (SouthernBiotech; 0100‐20) and images were taken with a fluorescent inverted microscope (Nikon Eclipse Ti2) and modified with the 64‐bit NIS Elements AR Analysis 5.360.02 analysis software and NIS Elements AR 5.360.02 image capturing software. Colocalization of VEGFR2 and EEA1 fluorescence was analyzed using JaCoP (Just another Colocalization) ImageJ plug‐in, which creates a mask over the VEGFR2 and EEA1 fluorescence signals and calculates Manders' coefficients (M1 and M2), which imply the actual overlap of the signals (A over B and B over A, respectively), and represent the true colocalization degree (Zinchuk et al., 2007). M1 and M2 coefficients were scored from 0 to 1 [e.g., M1 = 1.0 and M2 = 0.7, in red (signal A)‐green (signal B) pair, indicates that 100% of red pixels colocalize with green, and 70% of green pixels colocalize with red]. The M1 and M2 coefficients values were then multiplied by the percentage area of VEGFR2, accordingly, and plotted.

### 2.20 Proinflammatory panel MSD

The levels of proinflammatory cytokine expression were assessed using a V‐PLEX proinflammatory panel 1 (human) kit from MesoScale Discovery (MSD), which is able to measure the expression of 10 cytokines per sample. HCMECs were seeded and following 24 h treated with 25 μM AβQ22, 1 mM Hcy, or a combination of both for 3 h. Following treatment, media was collected and stored at −80°C until the MSD was conducted. The MSD was carried out according to the manufacturer's recommendations and the protein concentration was measured to normalize each sample.

### 2.21 Actin polymerization assay

The capability of cells to polymerize actin was assessed with an Actin Polymerization/Depolymerization Assay Kit (Abcam) according to the manufacturer's recommendations. HCMECs were seeded and following 24 h treated with 10 μM AβQ22, 1 mM Hcy, or a combination of both for 48 h. Following treatment, cells were lysed in a non‐denaturing buffer containing 20 mM Tris–HCl (pH 7.5) and 20 mM NaCl and homogenized with a glass dounce and cell extracts were immediately utilized for the assay. The fluorescence of each sample was read kinetically for 1 h and the change in fluorescent signal was calculated by subtracting the final read from the initial read. The percentage activation effect was calculated based on the kit's recommendations (percentage activation effect = (ΔSample/ΔPositive Control) × 100).

### 2.22 Statistical analysis

All experimental graphs are representative of at least three independent experiments with two or more technical duplicates. Data are represented as means±SEM. Statistical significance was assessed by one‐way ANOVA followed by Tukey's posthoc test using GraphPad Prism 9. Statistically significant differences required a *p* ≤ 0.05.

## 3 RESULTS

### 3.1 Hhcy potentiates Aβ‐mediated activation of the DR4 and DR5‐mediated extrinsic apoptotic pathway in HCMECs

**FIGURE 1:**
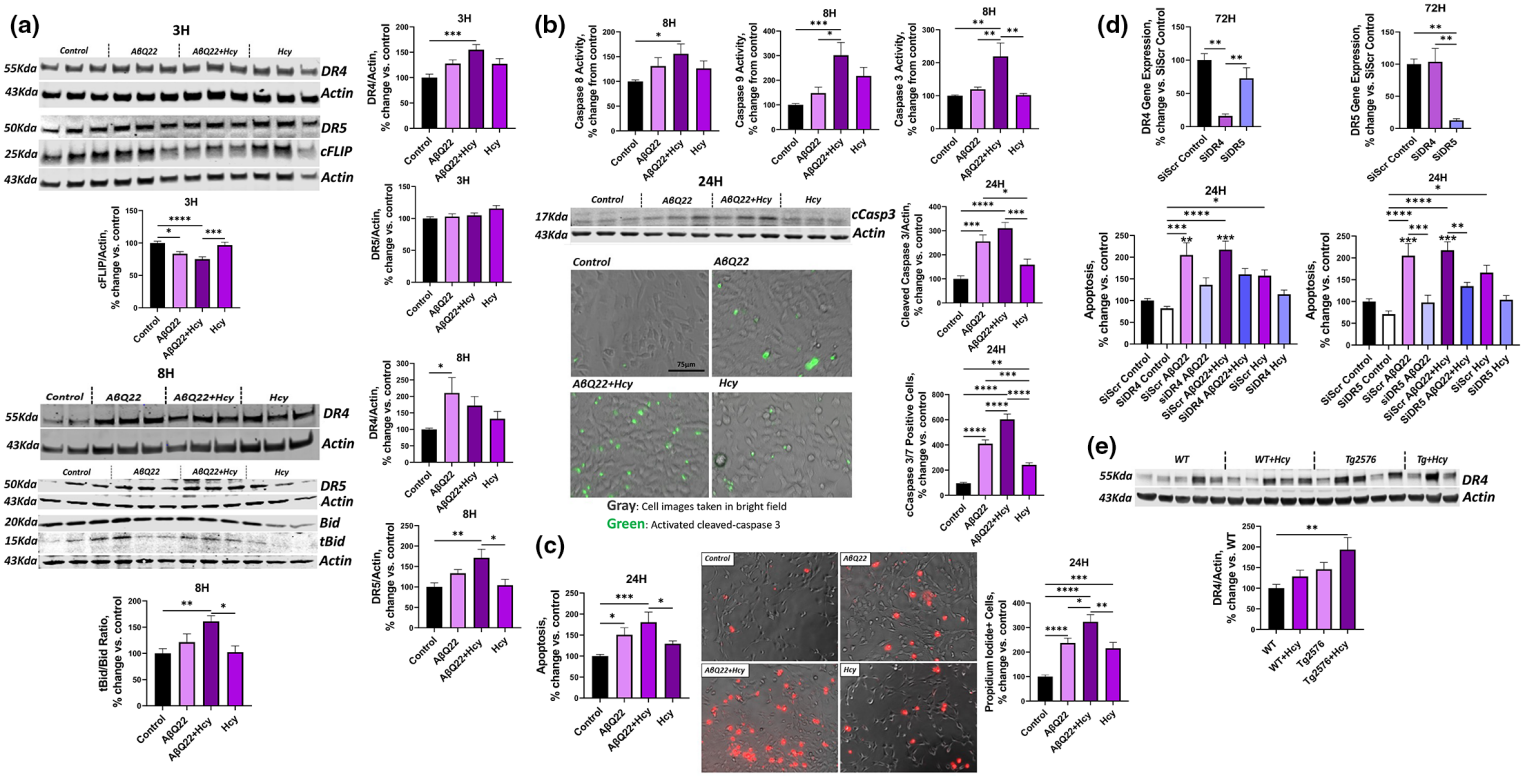
Hcy exacerbates AβQ22‐mediated DR4 and DR5 upregulation and potentiates activation of the extrinsic apoptotic pathway in HCMECs. (a) HCMECs were treated with 25uM AβQ22, 1 mM Hcy, or a combination of the two for 3 h or 8 h. DR4 (3/8 h), DR5 (3/8 h), cFLIP (3 h), and tBid/Bid (8 h) protein expression was evaluated via western blot. Actin was used for normalization and data is represented as % change vs. control. (b) HCMECs were treated with AβQ22, 1 mM Hcy, or a combination of the two for 8 h. Caspase 8, Caspase 9, and Caspase 3/7 activity was assessed via Promega's Caspase 3/7, 8, and 9 Glo Assays. Data is represented as % change vs. control. HCMECs were treated with 25uM AβQ22, 1 mM Hcy, or a combination of the two for 24 h. Cleaved caspase 3 protein expression was evaluated via western blot. Actin was used for normalization and data is represented as % change versus control. The amount of cleaved caspase 3/7 present in cells was evaluated with Thermo Fischer's Cell Event Caspase 3/7 fluorescence assay (imaged with Invitrogen's EVOS M5000 microscope). The % of caspase 3/7 positive cells was calculated as (caspase 3/7 positive cells/total cell number)*100. Data is represented as % change vs. control. (c) HCMECs were treated with 25uM AβQ22, 1 mM Hcy, or a combination of Hcy and AβQ22 for 24 h. Apoptosis was assessed as fragmented nucleosomes via a Cell Death ELISA (CDE). Data is represented as % change vs. control. HCMECs were treated with 25uM AβQ22, 1 mM Hcy, or a combination of the two for 24 h. The amount of propidium iodide positive cells was evaluated with a live cell propidium iodide stain imaged with Invitrogen's EVOS M5000 microscope and the % of propidium iodide positive cells was calculated as (propidium iodide positive cells/total cell number)*100. Data is represented as % change vs. control. (d) DR4 and DR5 silencing was confirmed by PCR and silencing was achieved at 72 h. HCMECs subjected to DR4 or DR5 silencing, or scrambled siRNA (SiScr) as a control, were treated with 25uM AβQ22, 1 mM Hcy, or a combination of Hcy and AβQ22 for 24 h. Apoptosis was assessed as fragmented nucleosomes via a CDE. Data is represented as % change vs. untreated control. For cell experiments in (a–d), *N* = 3 experiments with two or more technical replicates; one‐way ANOVA, Tukey's post‐test. (e) Western blot analysis of DR4 protein Expression in WT and Tg2576 mice ± Hhcy. Actin was used for normalization and data is represented as % change vs. control. *N* = 3–5 mice per group; *n* = 2 technical replicates. One‐way ANOVA, Tukey's post‐test. (**** *p* < 0.0001, *** *p* < 0.001, ** *p* < 0.01, * *p* < 0.05).

Aβ40 and its' vasculotropic variants have previously been shown to activate TRAIL DR‐mediated apoptosis in human cerebral microvascular ECs. In particular, we have shown that AβQ22 upregulates DR4 and DR5 expression and induces activation of the DRs‐mediated extrinsic apoptotic pathway, similarly but more rapidly than Aβ40‐WT (Fossati et al., 2012b). Hcy has been previously demonstrated to promote cell death in peripheral ECs through DRs, such as Fas (Suhara et al., 2004) (Tyagi et al., 2006). However, it's currently unknown whether Hcy activates TRAIL DR‐mediated apoptotic pathways in CECs. To determine whether high Hcy triggers EC death through activation of the same DR4/5‐mediated apoptotic pathway and whether combined challenge of HCMECs with AβQ22 and Hcy potentiates the activation of this specific apoptotic pathway, we conducted a time course analysis of DR4 and DR5 protein expression, which is known to be associated to their activation (Micheau et al., 1999; Poh et al., 2007; Rossin et al., 2009). HCMECs were treated with 25 μM AβQ22, 1 mM Hcy (concentrations previously shown to induce apoptosis in ECs) or a combination of the two for 3 h and 8 h (time points previously shown to induce DR4 and DR5 upregulation, respectively, in AβQ22‐challenged cerebral ECs) (Fossati et al., 2012b; Suhara et al., 2004). We observed that AβQ22 and Hcy upregulated DR4/5 expression at different time points. Combined treatment with Hcy and AβQ22 potentiated DR4 and DR5 upregulation, resulting in a significant increase compared to control cells at 3 h for DR4 and at 8 h for DR5 (Figure 1a). These results suggest that a combined challenge of HCMECs with AβQ22 and Hcy additively upregulates these DRs expression, with DR4 upregulation occurring earlier in time than DR5.

Activation of DRs, such as DR4 and DR5, directly induces caspase 8 activation (Cullen & Martin, 2009). cFLIP is an endogenous protein inhibitor of caspase 8 (Kataoka, 2005). Thus, a downregulation in cFLIP promotes caspase 8 activation, allowing for the progression of the extrinsic apoptotic pathway. To determine whether Hcy itself downregulates cFLIP expression and whether combined challenge with AβQ22 and Hcy additively decreases cFLIP, HCMECs were challenged with 25 μM AβQ22, 1 mM Hcy, or a combination of the two for 3 h. cFLIP protein expression was evaluated via western blot. Hcy itself did not significantly downregulate cFLIP expression. However, Hcy co‐treatment potentiated the downregulation of cFLIP‐induced by AβQ22 in HCMECs (Figure 1a).

Active caspase 8 promotes the cleavage of Bid into activated tBid, which is responsible for the recruitment of the intrinsic, mitochondria‐mediated apoptotic pathway, resulting in mitochondrial CytC release, caspase 9, and eventually caspase 3 activation (Fossati et al., 2012b). Previously, our lab has demonstrated a rapid increase in caspase 8 and 9 activity after an 8 h challenge with AβQ22 (Fossati et al., 2012b). To determine whether Hcy promotes similar caspase 8, 9 and 3 activations within cerebral ECs and whether combined challenge of HCMECs with AβQ22 and Hcy potentiates activation of these caspases, we conducted luminescent caspase‐glo activity assays at the 8 h timepoint. HCMECs were treated with AβQ22, Hcy, or a combination of the two for 8 h. HCMECs treated with AβQ22 and Hcy showed significant caspase 8, 9, and 3 activation, while activation by each challenge alone did not reach significance. Particularly, caspase 3 activation at 8 h was synergistically increased by the two challenges (Figure 1b). Since activation of caspase 8 induces Bid cleavage into tBid, Bid and tBid protein expression was evaluated by WB in HCMECs treated with 25 μM AβQ22, 1 mM Hcy, or a combination of the two for 8 h. The ratio of tBid to Bid (both proteins normalized to actin) was significantly higher in HCMECs treated with both AβQ22 and Hcy (Figure 1a), indicating that the combination treatment promotes Bid cleavage into tBid. The activation of caspase 3 was still potentiated in HCMECs treated with AβQ22 and Hcy for 24 h, as assessed via both western blot analysis and a fluorescent cleaved caspase 3 assay (Figure 1b). DNA fragmentation, indicative of the terminal stages of apoptosis, was also measured by the Cell Death ELISA^PLUS^ assay in HCMECs treated with 25 μM AβQ22, 1 mM Hcy, or a combination of the two for 24 h. Combined treatment with AβQ22 and Hcy additively potentiated DNA fragmentation compared to each challenge alone (Figure 1c). To confirm that the potentiation of apoptosis levels induced by AβQ22 and Hcy within HCMECs is specifically DR4 and DR5 mediated, HCMECs underwent DR4 and DR5 silencing and silencing efficiency was confirmed at 72 h post‐siRNA transfection via PCR (Figure 1d). As expected, scrambled siRNA transfected HCMECs treated with 25 μM AβQ22, 1 mM Hcy, or a combination of the two for 24 h demonstrated significantly increased levels of DNA fragmentation (Figure 1d). In contrast, DR4 and DR5 silencing significantly decreased HCMEC apoptosis levels in the face of these challenges, with DR5 silencing appearing to more robustly protect against AβQ22‐ and Hcy‐induced apoptosis. These results provide further evidence that AβQ22 and Hcy both operate through the DR‐mediated apoptotic pathway to promote apoptosis within HCMECs (Figure 1d). Additionally, necrosis was evaluated using a PI staining after the same 24 h challenges. Treatment of HCMECs with AβQ22 or Hcy independently increased PI levels compared to control cells and the combined treatment of HCMECs with AβQ22 and Hcy revealed a significant potentiation of necrosis compared to each challenge alone (Figure 1c).

To confirm in vivo whether Hhcy potentiates Aβ‐mediated upregulation of DR4, western blot analysis of DR4 expression was performed in the brain of WT and Tg2576 mice previously subjected from 5 to 13 months of age to a Hhcy‐inducing diet, or a control diet. WT mice with Hhcy and Tg2576 mice demonstrated a trend towards increased DR4 expression within the prefrontal cortex, while Tg2576 mice with Hhcy showed a significant upregulation of DR4, confirming the additive effects observed in vitro (Figure 1e). To note, unlike humans, the mouse genome expresses a single TRAIL death receptor (TRAIL‐R) that mimics and is homologous to both human DR4 and DR5, and is recognized as DR4 (Finnberg et al., 2008).

### 3.2 Hhcy exacerbates Aβ‐mediated mitochondrial apoptotic mechanisms in HCMECs

**FIGURE 2:**
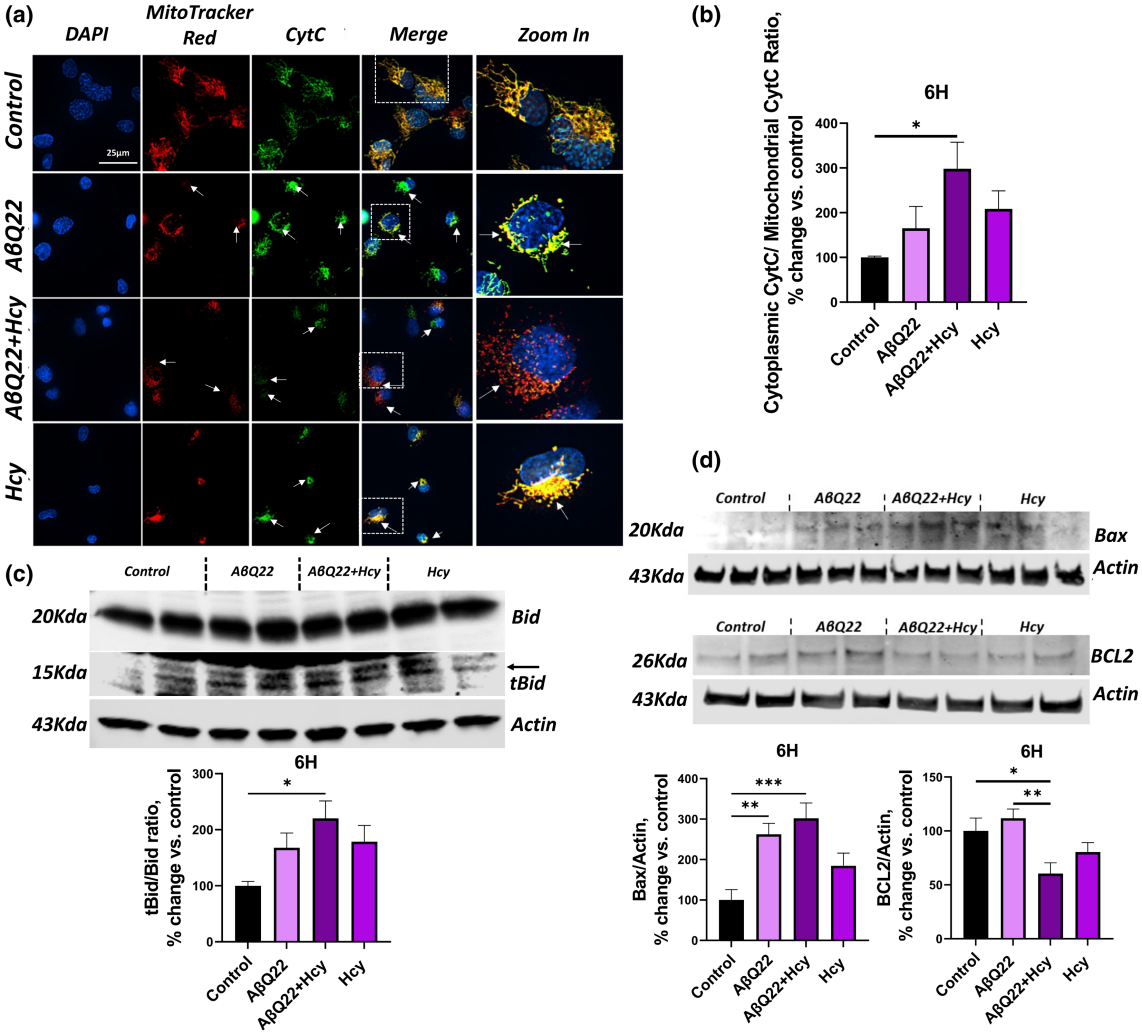
AβQ22 + Hcy treatment potentiates cytochrome C release, pro‐apoptotic Bax overexpression, and decreased anti‐apoptotic BCL2 expression in HCMECs. (A/B) HCMECs were treated with 25uM AβQ22, 1 mM Hcy, or a combination of the two for 6 h and Cytochrome C (CytC) release was evaluated by immunocytochemistry (a) and ELISA (b). (a) HCMECs were stained with DAPI (blue), MitotrackerRed (red), and CytC (green). (b) Following treatment, mitochondrial and cytoplasmic extracts were obtained and utilized for a CytC ELISA. Data is represented as the ratio of cytoplasmic CytC signal to mitochondrial CytC signal (% change vs. control). (c) HCMECs were treated with 25uM AβQ22, 1 mM Hcy, or a combination of the two for 6 h. tBid/Bid protein expression ratio was evaluated via western blot and actin was used for normalization. Data is represented as % change versus control. (d) HCMECs were treated with 25uM AβQ22, 1 mM Hcy, or a combination of the two for 6 h. Bax and BCL2 protein expression were evaluated via western blot and actin was used for normalization. Data is represented as % change vs. control. For (a–d), N ≥ 3 experiments with two or more technical replicates; one‐way ANOVA and Tukey's post‐test. (****p < 0.0001, ***p < 0.001, **p < 0.01, *p < 0.05).

AβQ22 was previously shown to promote release of CytC from the mitochondria to the cytoplasm in cerebral ECs (Fossati et al., 2010; Solesio et al., 2018). Hcy has also been found to promote CytC release in peripheral ECs (Tyagi et al., 2006). To determine whether Hcy alone promotes CytC release in cerebral ECs and to assess whether combined challenge with AβQ22 and Hcy potentiates CytC release, we conducted CytC immunocytochemistry and ELISA assays after 6 h treatments. As shown in Figure 1b, AβQ22 and Hcy treatment of HCMECs resulted in a synergistic increase in caspase 3 activity at 8 h, therefore, since CytC release precedes caspase 3 activation, CytC release was measured after 6 h challenge. Following treatment, HCMECs were stained with DAPI (blue), MitoTracker Red CM‐H_2_XRos (red), and CytC (green). CytC staining in control HCMECs highlighted, as expected, chain‐like mitochondrial structures, which colocalized with MitoTracker Red CM‐H_2_XRos, a dye that enters the mitochondria upon a healthy membrane potential (Figure 2a). HCMECs treated with AβQ22 demonstrated a partial loss of CytC chain structure, perinuclear localization of mitochondria, and a loss of mitochondrial membrane potential in some of the cells. Similar effects were induced by treatment with Hcy (Figure 2a). However, when HCMECs were treated with a combination of AβQ22 and Hcy, a complete loss of mitochondrial CytC chain structure was observed, with significant evidence of mitochondrial fragmentation and most of the CytC signal was lost, suggesting that CytC had been more rapidly released from the mitochondria into the cytoplasm (Figure 2a). To confirm this phenomenon, following treatment, a mitochondrial isolation was conducted to obtain mitochondrial and cytoplasmic fractions from HCMECs treated with AβQ22, Hcy, or the combination, for 6 h. For each sample, CytC levels were measured in mitochondrial and cytoplasmic fractions using a CytC ELISA and the ratio of CytC in the cytoplasm vs. CytC in the mitochondria was calculated, with a higher ratio being indicative of more cytoplasmic CytC and less mitochondrial CytC. HCMECs treated with either AβQ22 or Hcy alone demonstrated trends towards increased CytC release into the cytoplasm, but when ECs were treated with the combination of AβQ22 and Hcy, a significantly higher CytC release was observed (Figure 2b). Truncation of Bid into tBid is an event known to promote CytC release. Thus, to determine if the observed CytC release was tBid‐mediated, Bid and tBid protein expression was also evaluated at 6 h in HCMECs treated with AβQ22, Hcy, or the combination (Figure 2c). The ratio of tBid to Bid (both proteins normalized to actin) was significantly higher in HCMECs treated with both AβQ22 and Hcy, mirroring the results from the 8 h timepoint (Figure 1a), suggesting that the combination treatment promotes potentiated Bid cleavage into tBid and thus exacerbated release of CytC (Figure 2c). Bax and BCL2 protein expression, whose equilibrium mediates mitochondrial voltage‐dependent anion channel opening and CytC release, were also measured in the same conditions. HCMECs treated with AβQ22 for 6 h demonstrated a significant upregulation of the pro‐apoptotic protein Bax, while HCMECs treated with Hcy demonstrated a trend towards increased Bax protein expression (Figure 2d). In HCMECs treated with a combination of AβQ22 and Hcy, however, the increase in Bax expression was the most significant compared to control conditions (Figure 2d). Additionally, BCL2 expression was significantly decreased in HCMECs treated with a combination of AβQ22 and Hcy for 6 h (Figure 2d).

### 3.3 Hhcy intensifies Aβ‐mediated deregulation of BBB junction proteins and loss of barrier resistance

**FIGURE 3:**
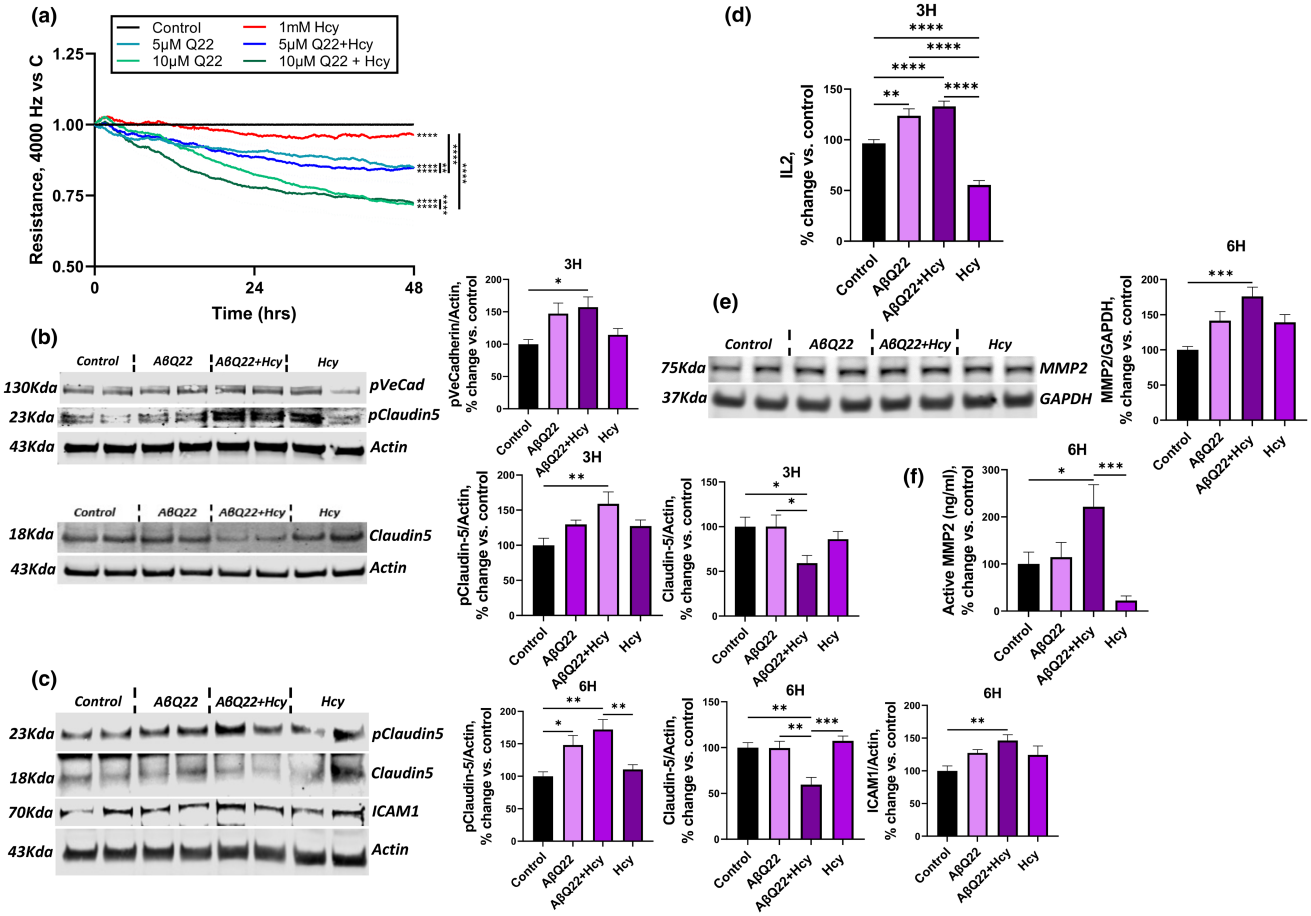
AβQ22 + Hcy challenge potentiates decrease in barrier resistance, tight junction protein dysfunction, and increases in ICAM, MMP2, and IL2. (a) Trans‐endothelial electrical resistance was monitored for 48 h post‐barrier formation with the ECIS Zθ system (Applied Biophysics). After formation of a HCMEC‐barrier, cells were treated with 5 or 10 uM AβQ22, 1 mM Hcy, or a combination of the two. Data are represented as resistance change versus control (black line) (*N* = 3 experiments with two technical replicates per group; one‐way ANOVA). (b) HCMECs were treated with 25uM AβQ22, 1 mM Hcy, or a combination of the two for 3 h. Phosphorylated VE‐cadherin, claudin5, and phosphorylated claudin5 protein expression were evaluated via western blot and actin was used for normalization. Data is represented as % change versus control (*N* ≥ 3 experiments with two or more technical replicates; one‐way ANOVA; Tukey's post‐test). (c) HCMECs were treated with 25uM AβQ22, 1 mM Hcy, or a combination of the two for 6 h. Claudin5, phosphorylated claudin5, and ICAM protein expression was evaluated via western blot and actin was used for normalization. Data is represented as % change versus control (*N* ≥ 3 experiments with two or more technical replicates; one‐way ANOVA; Tukey's post‐test). (d) HCMECs were treated with 25uM AβQ22, 1 mM Hcy, or a combination of the two for 3 h and media was collected to run a multiplex inflammatory cytokine assay (MSD) and protein concentration was used for normalization. Data is represented as % change versus control (*N* ≥ 3 experiments with two or more technical replicates; one‐way ANOVA; Tukey's post‐test). (e/f) HCMECs were treated with 25uM AβQ22, 1 mM Hcy, or a combination of the two for 6 h. (e) MMP2 protein expression was evaluated via western blot and GAPDH was used for normalization. (f) MMP2 activity was evaluated with an MMP2 activity assay (QuickZyme Biosciences). Data is represented as % change versus control (*N* ≥ 3 experiments with two or more technical replicates; one‐way ANOVA; Tukey's post‐test). (**** *p* < 0.0001, *** *p* < 0.001, ** *p* < 0.01, * *p* < 0.05).

We have recently shown that AβQ22 decreases TEER of cerebral EC monolayers (Parodi‐Rullan et al., 2020). Hcy has also been demonstrated to affect BBB integrity (Beard Jr. et al., 2011; Kamath et al., 2006; Rhodehouse et al., 2013). To determine whether combined treatment of HCMECs with AβQ22 and Hcy additively diminishes barrier resistance, ECIS technology was utilized to monitor TEER over time. Following barrier formation, HCMECs were treated with sublethal concentrations of AβQ22 (5 μM or 10 μM), 1 mM Hcy, or a combination of the two and TEER was monitored for 48 h. Cells treated with AβQ22 or Hcy separately demonstrated a significant decrease in TEER **(**Figure 3a **)**. Moreover, when cells were treated with the combination of AβQ22 (both the 5 and 10 μM) and Hcy, an additive decrease in TEER was observed **(**Figure 3a **)**, indicative of an additively increased BBB permeability. To understand whether early changes in the expression of junction proteins and other mediators of barrier permeability are responsible for Hcy and AβQ22‐induced loss of barrier resistance, we investigated the expression of TJ proteins and other proteins known to regulate EC barrier permeability at early (3 h and/or 6 h) timepoints, which are prior to substantial losses in TEER after challenge. Western blot was utilized to evaluate known mediators of endothelial barrier permeability (Hatanaka et al., 2011; Sidibe & Imhof, 2014; Yamamoto et al., 2008), such as phosphorylated VE‐cadherin, claudin5, and phosphorylated claudin5 expression, in HCMECs treated with 25 μM AβQ22, 1 mM Hcy, or the combination, for either 3 h or 6 h. At 3 h, the combination of AβQ22 and Hcy significantly potentiated the increase in phosphorylated VE‐cadherin, an adherent protein that upon phosphorylation causes ECs to retract from one another (Hatanaka et al., 2011; Sidibe & Imhof, 2014) **(**Figure 3b **)**. Also, after 3 h treatment with AβQ22 and Hcy, ECs revealed a significant reduction in claudin5 expression compared to controls and Aβ treatment alone **(**Figure 3b **)**, which was maintained at 6 h (Figure 3c). The combination treatment also induced an additive increase in phosphorylated claudin5 at 3 h and 6 h, which has been associated with the loss of BBB integrity (Yamamoto et al., 2008) (Figure 3b/c). To evaluate possible mediators of immune cell adhesion, the expression of ICAM (Intercellular Adhesion Molecule 1), a protein expressed by ECs to promote immune cell extravasation, was also investigated in HCMECs treated with 25 μM AβQ22, 1 mM Hcy, or the combination, for 6 h. Cells treated with the combination treatment demonstrated a significant increase in ICAM expression compared to controls (Figure 3c). Since ICAM upregulation is typically mediated by immune activation through cytokine production, we asked whether cytokine expression preceded ICAM overexpression in our cells. A multiplex cytokine assay (MSD) performed on media from HCMECs treated with AβQ22, Hcy, or the combination for 3 h, revealed that, while Hcy alone decreased IL2 levels, AβQ22 alone and the combination of AβQ22 and Hcy induced a significant increase in IL2 expression, with the combination treatment inducing the most significant increase (Figure 3d). Other pro‐inflammatory cytokines tested were not significantly increased at this time point (Figure S1). In addition, the expression of MMP2, an enzyme responsible for breaking down extracellular matrix proteins like collagen, was explored in HCMECs treated with AβQ22, Hcy, or the combination of the two for 6 h (Wang et al., 2020). Cells treated with both AβQ22 and Hcy demonstrated a significant and additive increase in MMP2 expression compared to controls (Figure 3e). Moreover, we observed a synergistical increase in MMP2 activity when cells were challenged with the combined treatment (Figure 3f). Overall, these changes in specific functional components of the cerebral endothelial barrier explain the severe loss in TEER observed in HCMECs treated with the combination treatment.

**FIGURE 4:**
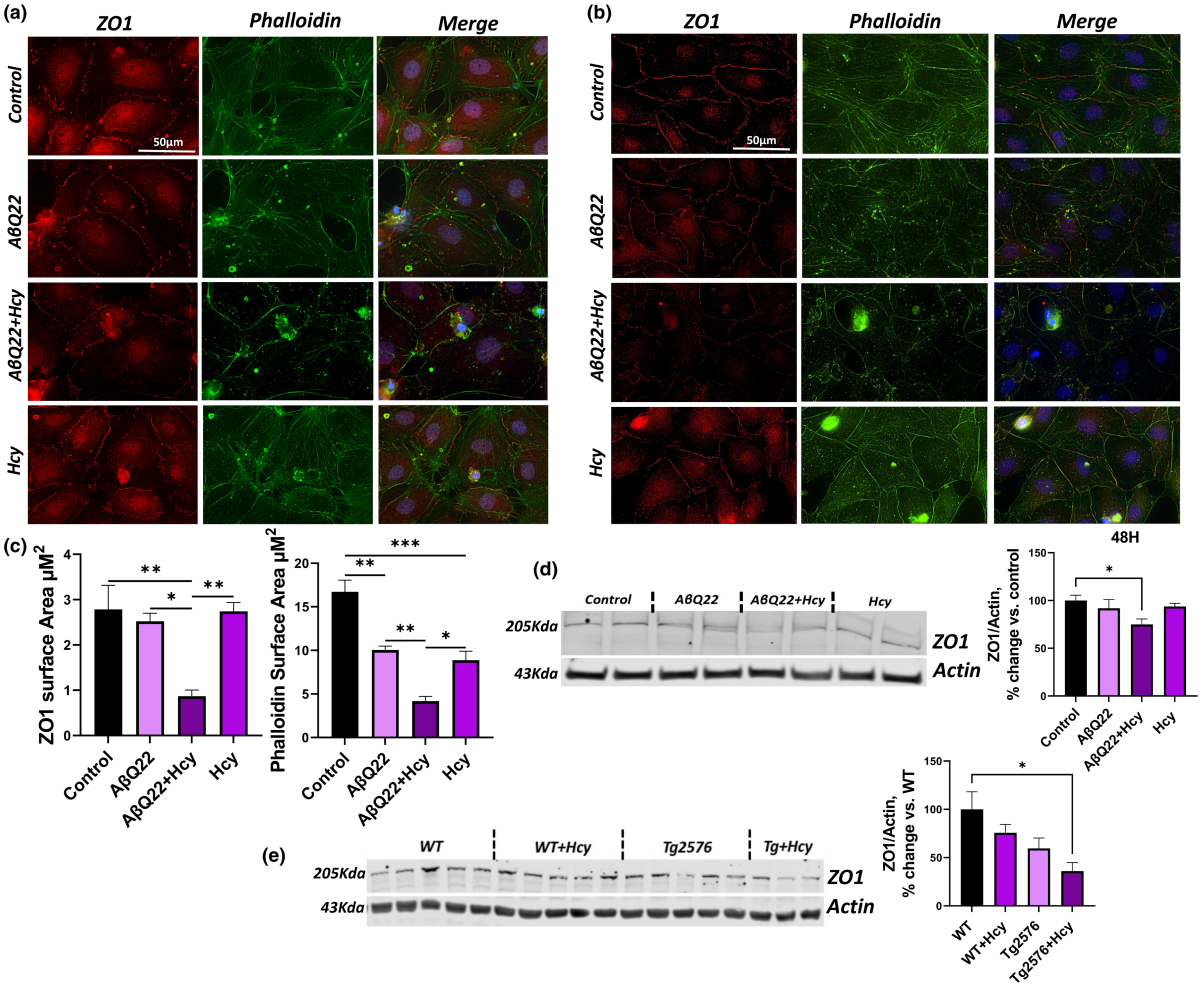
Hhcy potentiates AβQ22‐mediated loss of ZO1 in human cerebral endothelial cells and in the mouse cortex. (a/b) Primary human cerebral endothelial cells were treated with 10uM AβQ22, 1 mM Hcy, or a combination of the two either prior to barrier formation (a) or post‐barrier formation (b). ZO1 and phalloidin expression were evaluated by immunocytochemistry. Primary human cerebral endothelial cells were stained with DAPI (blue), ZO1 (red), and phalloidin (green). (c) Fluorescence of ZO1 and phalloidin in panel (b) were quantified with the Halo software (Indica Labs). (d) HCMECs were treated with 10uM AβQ22, 1 mM Hcy, or a combination of the two for 48 h. ZO1 protein expression was evaluated via western blot and actin was used for normalization. Data is represented as % change versus control (*N* ≥ 3 experiments with two technical replicates; one‐way ANOVA; Tukey's post‐test). (e) Western blot analysis was utilized to assess ZO1 protein expression in WT and Tg2576 mice ± Hhcy. Actin was used for normalization and data is represented as % change versus control (*N* = 3–5 mice per group; *n* = 1 technical replicate; one‐way ANOVA, Tukey's post‐test). (**** *p* < 0.0001, *** *p* < 0.001, ** *p* < 0.01, * *p* < 0.05).

A dramatic decrease in TEER, indicative of severe EC barrier damage, was observed at 48 h, especially in HCMECs treated with the combination of AβQ22 and Hcy. Hence, the expression of TJ proteins was also assessed in HCMECs treated with 10 μM AβQ22, 1 mM Hcy, or a combination of the two for 48 h. The deregulation of ZO1 expression and membrane localization was assessed by immunocytochemistry. The cells were treated either prior to endothelial barrier formation (Figure 4a) or after establishment of the barrier (Figure 4b). During the process of EC barrier formation, control cells demonstrate a robust network of actin filaments (green) and clear evidence of ZO1 (red) expression between cells attaching to these actin filaments to anchor the cells closer together (Figure 4a). HCMECs treated with AβQ22 or Hcy individually demonstrated a derangement in the actin cytoskeleton and appeared to have a decreased connection of ZO1 to the actin filaments (Figure 4a). HCMECs treated with the combination of AβQ22 and Hcy demonstrated a loss of ZO1 expression and an even more extreme loss of structure in the actin cytoskeleton (Figure 4a). Once the barrier was established, control cells showed, as expected, a continuous outline of ZO1 bordering neighbor cells (Figure 4b). In HCMECs treated with AβQ22 or Hcy post‐barrier formation, noticeable interruptions in the ZO1 outline can be observed, as well as a loss in actin filaments, while HCMECs treated with the combination of AβQ22 and Hcy revealed an almost complete loss of the ZO1 outline, and a severe disruption of the actin filament network, further corroborated by the quantification of ZO1 and Phalloidin fluorescence intensity (Figure 4c). The proper cobblestone EC morphology was also severely altered by the combination treatment. Western blot analysis confirmed that ZO1 expression was significantly reduced by the combination treatment at this time point (Figure 4d).

To elucidate in vivo whether Hhcy contributes to the loss of ZO1 and potentiates Aβ‐mediated ZO1 decrease, western blot analysis of ZO1 expression was evaluated within the prefrontal cortex of WT and Tg2576 mice with or without Hhcy‐inducing diet. WT mice with Hhcy and Tg2576 mice demonstrated a trend towards decreased ZO1 expression. A significant and additive loss of ZO1 protein expression was observed in Tg2576 mice with Hhcy (Figure 4e), confirming our in vitro results.

### 3.4 Aβ and Hcy additively decrease angiogenic capabilities of HCMECs

**FIGURE 5:**
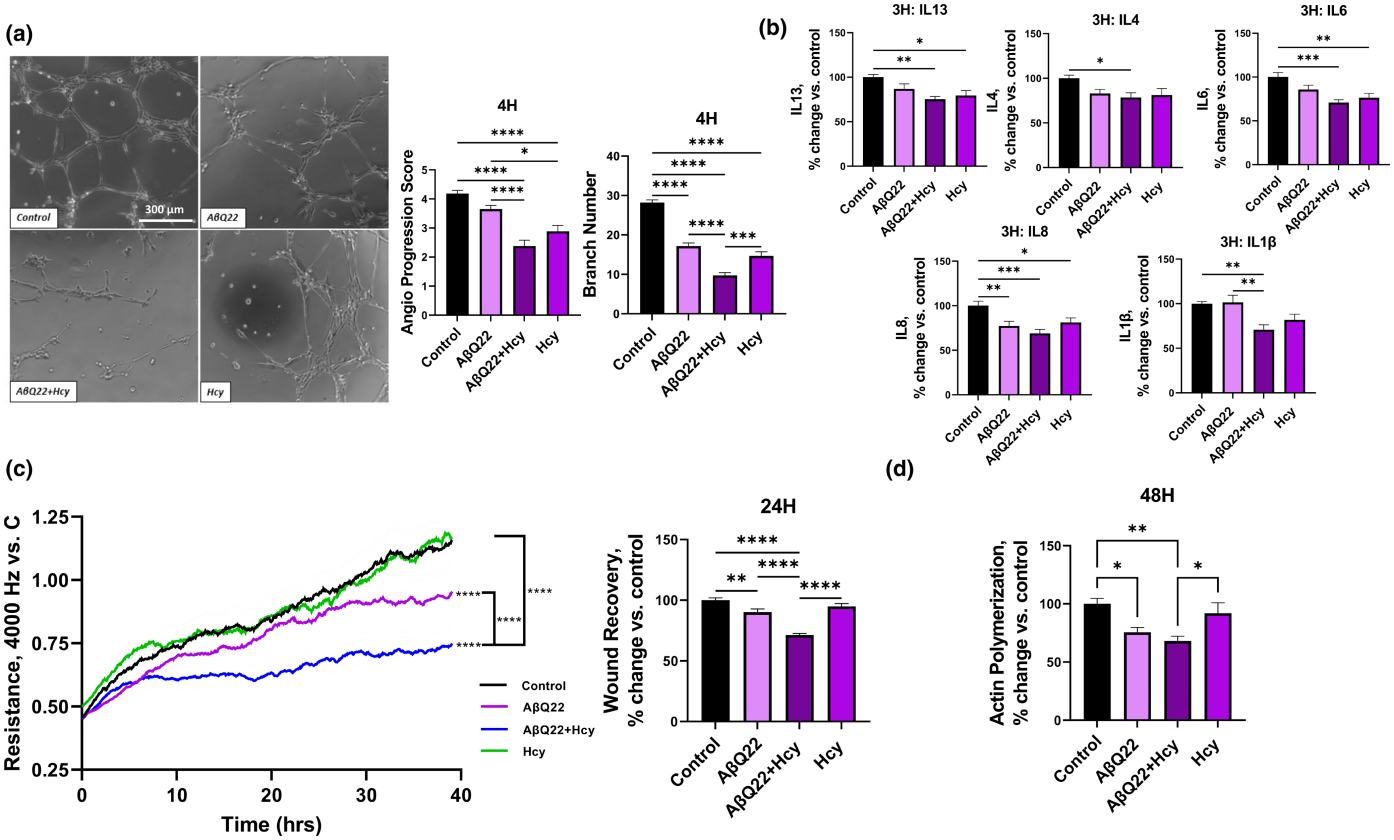
AβQ22 + Hcy potentiates decreases in angiogenic and wound healing capabilities, angiogenesis‐related cytokine expression, and Actin polymerization in HCMECs. (a) HCMECs were treated with 1uM AβQ22, 500uM Hcy, or a combination of the two for 4 h. Vessel branch number and angiogenesis progression score were assessed via an angiogenesis inhibition kit. Data is represented as % change versus control (*N* = 3 experiments with two or more technical replicates; one‐way ANOVA, Tukey's post‐test). (b) HCMECs were treated with AβQ22, Hcy, or a combination of the two for 3 h and media was collected to run a multiplex inflammatory cytokine assay (MSD) and protein concentration was used for normalization. Data is represented as % change vs. control (*N* ≥ 3 experiments with two or more technical replicates; one‐way ANOVA; Tukey's post‐test). (c) HCMEC would healing ability was assessed with an ECIS would healing assay. Post‐barrier formation, HCMECs were treated with 10uM AβQ22, 1 mM Hcy, or a combination of the two and 1 h post‐treatment were wounded for 20 s (60,000Hz). Following the wound, HCMEC trans‐endothelial electrical resistance was monitored and an increase in TEER post‐wound is indicative of wound healing. Data is represented as resistance change vs. control (black line) (*N* = 4 experiments with two technical replicates per group; one‐way ANOVA). The graph starting point is 1 h post‐treatment, when the wound was inflicted. TEER readings at 24 h were plotted in a bar graph. Data is represented as % change versus control (one‐way ANOVA, Tukey's post‐test). (d) HCMECs were treated with 10uM AβQ22, 1 mM Hcy, or a combination of the two for 48 h and the samples collected were utilized for an actin polymerization assay (Abcam). Data is represented as % change vs. control (*N* = 3 experiments with two or more technical replicates; one‐way ANOVA; Tukey's post‐test). For A‐D, **** *p* < 0.0001, *** *p* < 0.001, ** *p* < 0.01, * *p* < 0.05.

We have recently shown that Aβ peptides, including AβQ22, promote deficits in human cerebral EC angiogenesis (Parodi‐Rullan et al., 2020). In particular, AβQ22 significantly inhibited angiogenesis already at the concentration of 1 μM. Hcy has been observed to disrupt angiogenic capabilities of HUVEC ECs; however, it is not established if it can affect cerebral angiogenesis (Pan et al., 2017; Tian et al., 2020; Zhang et al., 2012). To determine whether Hcy promotes angiogenic deficits in cerebral ECs and whether combined challenge of HCMECs with AβQ22 and Hcy additively decreases HCMECs angiogenic capability, we performed an angiogenesis inhibition assay. ECs were treated with 1 μM AβQ22 (known as the lowest dose capable of inducing angiogenesis inhibition in this vessel formation assay in our previous studies), 500 μM Hcy, or the combination of the two for 4 h, the earliest timepoint by which we observed healthy control cells capable of establishing extensive vessel formation. Angiogenesis progression score and branch number of cerebral ECs were analyzed. Treatment with Hcy decreased angiogenesis progression score and branch number (Figure 5a). When HCMECs were treated with both AβQ22 and Hcy, an additive decrease in angiogenesis progression score and vessel branch number was observed (Figure 5a). Since particular cytokines are known to trigger angiogenesis, angiogenesis‐related cytokine release was assessed utilizing the same multiplex cytokine array from MSD as above. HCMECs were treated with AβQ22, Hcy, or a combination of the two for 3 h and media was collected. Since angiogenesis changes are apparent by 4 h, we sought to measure changes within angiogenesis‐related cytokine expression at 3 h, as these changes could influence the formation of stable vessels. The expression of IL13, IL4, IL8, IL1β, and IL6, which all have been previously found to play a role in the promotion of angiogenesis, were additively decreased in HCMECs treated with the combination of AβQ22 and Hcy (Figure 5b). Changes in other cytokines not specifically related to angiogenesis are presented in Figure S1. Moreover, to investigate whether the combination of Aβ and Hcy affects EC repair mechanisms, a wound healing assay was conducted using the ECIS technology. Following barrier formation, HCMECs were treated with 10 μM AβQ22, 1 mM Hcy, or a combination of the two and 1 h posttreatment, a circular wound was inflicted in the center of the EC barrier by a strong electrical current. Recovery from the wound, indicated by increases in TEER post‐wound, was monitored for 48 h. HCMECs treated with Hcy were able to recover up to the control level, while HCMECs treated with AβQ22 showed impairment in recovery ability, only reaching about half the recovery of the controls (Figure 5c). HCMECs treated with the combination of AβQ22 and Hcy were completely unable to recover from the wound, showing significant impairment in wound healing compared to controls and both treatments alone (Figure 5c). AβQ22 and Hcy affect the actin cytoskeleton in HCMECs at 48 h (Figure 4b/c). Since the polymerization of actin is essential for cell proliferation, wound healing and BBB formation, the ability of HCMECs to polymerize actin was also assessed in cells treated with AβQ22, Hcy, or the combination of the two for 48 h. HCMECs treated with the Aβ peptide demonstrated a significant decrease in actin polymerization compared to control cells and HCMECs treated with Hcy demonstrated a similar, but nonsignificant trend (Figure 5d). As expected, treatment with the combination of AβQ22 and Hcy resulted in the most significant decrease in actin polymerization capability (Figure 5d).

**FIGURE 6:**
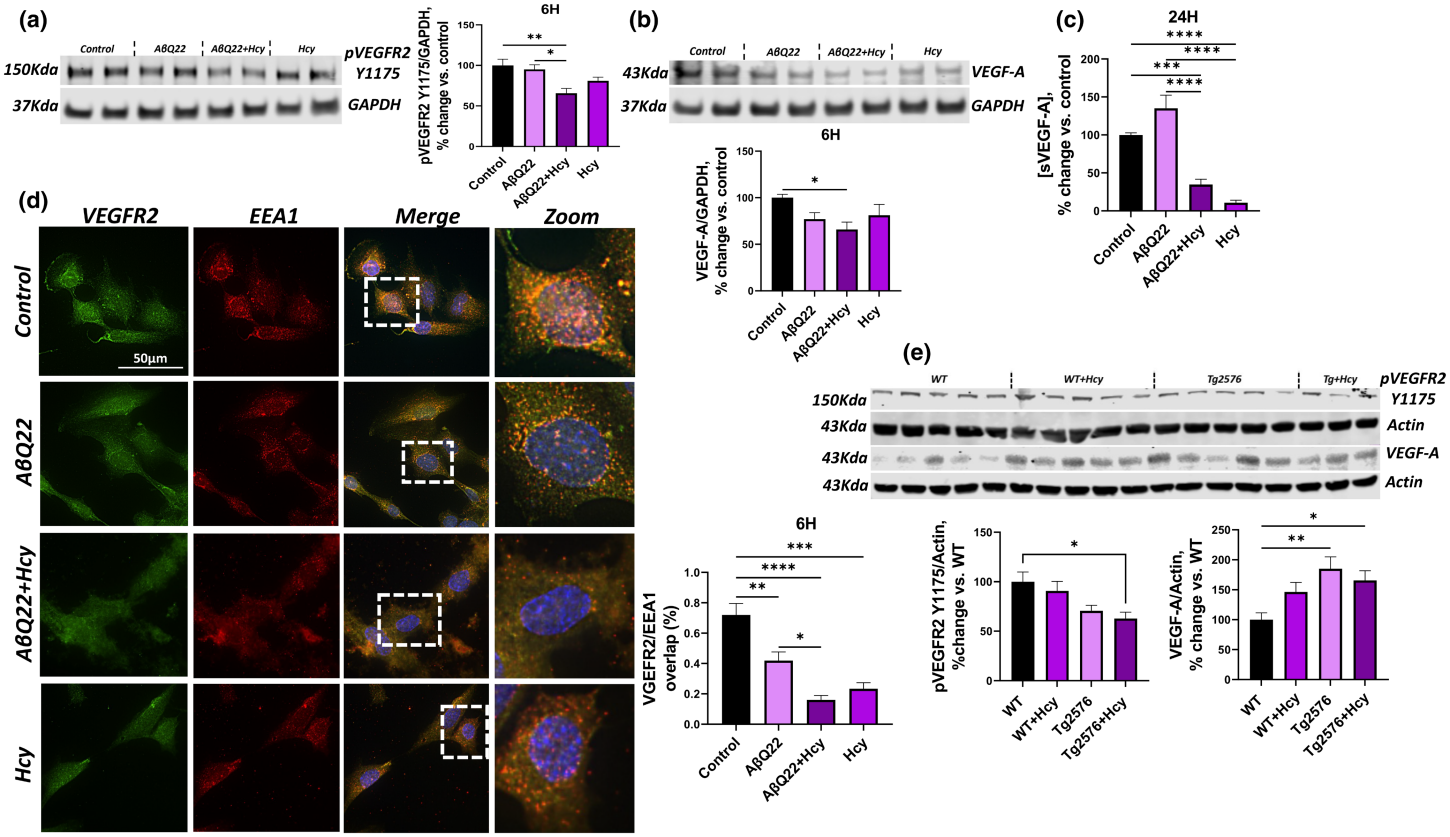
Hcy potentiates AβQ22‐mediated loss of angiogenic mediators and VEGFR2 internalization within early endosomes. (a) HCMECs were treated with 25uM AβQ22, 1 mM Hcy, or a combination of the two for 6 h. pVEGFR2 protein expression was evaluated via western blot and GAPDH was used for normalization. Data is represented as % change versus control (*N* = 3 experiments with two technical replicates; one‐way ANOVA; Tukey's post‐test). (b) HCMECs were treated with 25uM AβQ22, 1 mM Hcy, or a combination of the two for 6 h. VEGF‐A protein expression was evaluated via western blot and GAPDH was used for normalization. Data is represented as % change vs. control ((*N* = 3 experiments with two technical replicates; one‐way ANOVA; Tukey's post‐test). (c) HCMECs were treated with 25uM AβQ22, 1 mM Hcy, or a combination of the two for 24 h and media was collected to run a soluble VEGF‐A ELISA (sVEGF‐A). Data is represented as % change vs. control (*N* = 3 experiments with two or more technical replicates; one‐way ANOVA; Tukey's post‐test). (d) HCMECs were treated with 25uM AβQ22, 1 mM Hcy, or a combination of the two for 6 h and VEGFR2 internalization into early endosomes was evaluated by immunocytochemistry. HCEMCs were stained with DAPI (blue), VEGFR2 (green), and EEA1 (red). Colocalization of VEGFR2 fluorescence in EEA1 positive endosomes was analyzed with Halo software (Indica Labs). (e) Western blot analysis was utilized to assess pVEGFR2 and VEGF‐A protein expression in WT and Tg2576 mice −/+ Hhcy. Actin was used for normalization and data is represented as % change versus control (*N* = 3–5 mice per group; *n* = 2 technical replicates; one‐way ANOVA, Tukey's post‐test). For a–e: **** *p* < 0.0001, *** *p* < 0.001, ** *p* < 0.01, * *p* < 0.05.

Upon binding of VEGF‐A to the VEGFR2, the receptor is phosphorylated at tyrosine Y1175 to promote EC proliferation and angiogenesis. Vascular endothelial growth factor A (VEGF‐A) and phosphorylated vascular endothelial growth factor receptor 2 (VEGFR2) at tyrosine1175 (pVEGFR2 Y1175) protein expression was assessed by WB in HCMECs treated with AβQ22, Hcy, or a combination of both for 6 h. Following the combination treatment, we observed an additive decrease in both the expression of VEGF‐A (the VEGFR2 ligand), and in pVEGFR2 Y1175 (Figure 6a/b). To assess whether the combined treatment of HCMECs with AβQ22 and Hcy continues to inhibit the release of VEGF‐A at longer timepoints, soluble VEGF‐A levels were also analyzed by an ELISA assay in cell media at 24 h. Interestingly, HCMECs treated with Hcy or Aβ + Hcy released significantly lower levels of soluble VEGF‐A after 24 h compared to controls and Aβ‐treated cells **(**Figure 6c **)**.

Once the VEGFR2 is bound, the receptor dimerizes and is subsequently internalized within endosomes, where it is phosphorylated (Balaji Ragunathrao, Vellingiri, Anwar, Akhter, & Mehta, 2020). Internalization of the phosphorylated VEGFR2 into early endosomes protects the receptor from degradation and sustains the activation of signaling that promotes angiogenesis (Balaji Ragunathrao et al., 2020). To assess how AβQ22, Hcy, and the combination modulate VEGFR2 internalization into early endosomes, immunocytochemistry for VEGFR2 and EEA1 (early endosome antigen 1), was conducted in HCMECs treated with 25 μM AβQ22, 1 mM Hcy, or a combination of the two for 6 h. In control cells, a prevalent colocalization of VEGFR2 and EEA1 was observed (Figure 6d). In HCMECs treated with AβQ22 or Hcy, we detected a notable decrease in both VEGFR2 and EEA1 staining, as well as a decrease in colocalization of VEGFR2 and EEA1. When HCMECs were treated with the combination of AβQ22 and Hcy, an almost complete loss of VEGFR2 and EEA1 expression was observed, as well as a severe decrease in VEGFR2 and EEA1 colocalization (Figure 6d). To confirm whether Hhcy contributes to decreased pVEGFR2 Y1175 and potentiates Aβ‐mediated pVEGFR2 Y1175 decreases in vivo, western blot analysis of pVEGFR2 Y1175 was evaluated in the prefrontal cortex of WT and Tg2576 mice subjected or not to Hhcy‐inducing diet. WT mice with Hhcy and Tg2576 mice demonstrated a trend towards decreased pVEGFR2 Y1175 expression. Importantly, as hypothesized, a significant and additive loss of pVEGFR2 Y1175 protein expression was observed in Tg2576 mice with Hhcy (Figure 6e). VEGF‐A protein expression was also assessed within the same prefrontal cortex samples. Interestingly, VEGF‐A was significantly upregulated in Tg2576 mice with and without Hhcy (Figure 6e). These results suggest that, even though Tg2576 mice seem to be overexpressing VEGF‐A as a compensatory mechanism, there is maladaptive VEGFR2 activation, evidenced by the observation that Tg2576 mice, particularly in the presence of Hhcy, have significantly decreased levels of the activated pVEGFR2, which represents the direct result of VEGF‐A/VEGFR2 binding.

## 4 DISCUSSION

It is well accepted that CV risk factors, including Hhcy, increase the risk and pathological severity of AD (Carey & Fossati, 2023; Kamat et al., 2015; Tinelli et al., 2019). Previous studies demonstrated that Hhcy accelerates cerebral amyloidosis and CAA, exacerbating deficits in CBF and cerebrovascular dysfunction in AD mouse models (Braun et al., 2019; Li et al., 2014; Li & Pratico, 2015; Sudduth et al., 2013; Zhuo et al., 2010; Zhuo & Pratico, 2010). Additionally, neuroinflammatory processes, microhemorrhages and cerebrovascular atherosclerosis were observed in postmortem human brains of patients with Hhcy (Weekman et al., 2022). It is evident that Hhcy has severe pathological consequences on the cerebrovascular environment. However, the specific endothelial molecular mechanisms responsible for these deleterious effects and for the combined impact of Hhcy and amyloid cerebrovascular pathology remained poorly elucidated (Carey & Fossati, 2023). This study demonstrates that a combination challenge of HCMECs with a vasculotropic Aβ peptide, the Dutch mutant, and Hcy exacerbates TRAIL DR4/5‐mediated extrinsic apoptosis, BBB permeability, and angiogenesis impairment. Importantly, we described specific concurrent molecular mechanisms through which both AβQ22 and Hhcy act to induce brain EC dysfunction, and we observed that the combined effect of these two cerebrovascular challenges on cerebral ECs is, in most cases, additive in nature. The AβQ22 peptide has been selected for this study because of the specific association of this mutation with CAA, vascular abnormalities and cerebrovascular dysfunction in human Dutch familial cases (Hereditary cerebral hemorrhage with amyloidosis Dutch type) (Fossati et al., 2010; Frangione et al., 2001; Ghiso & Frangione, 2001; Levy et al., 1990). Our previous studies showed that Aβ40‐WT, the Aβ peptide that preferentially deposits on the vasculature in sporadic AD, has analogous effects in longer times, once similar aggregation species develop (Fossati et al., 2010, 2012b).

Cerebral EC apoptosis contributes to the pathogenesis of AD, sporadic and familial forms of CAA (Take et al., 2022); Wang, Zhang, et al., 2020), being one of the main culprits for the appearance of micro and macro‐hemorrhages (Wang et al., 2020). We have previously shown that the vasculotropic Dutch Aβ mutant, AβQ22, highly associated with CAA, promotes, TRAIL DRs (DR4/5)‐mediated cerebral EC apoptosis (through direct binding to these receptors), BBB permeability, and angiogenesis deficits similarly to Aβ40‐WT, albeit with faster kinetics (Fossati et al., 2010, 2012b; Parodi‐Rullan et al., 2020, 2021). Therefore, this peptide represents an excellent tool to study the effects of Aβ‐mediated endothelial pathology in vitro. Multiple studies have also demonstrated that Hcy promotes cerebral EC dysfunction (Faraci & Lentz, 2004; Kamat et al., 2015; Lai & Kan, 2015; Suhara et al., 2004; Tyagi et al., 2006). However, it is not known if Hcy can induce DR4/5‐mediated EC apoptosis.

Our current study is the first to demonstrate that high Hcy potentiates the toxic effects of Aβ on cerebral EC through the DR4/5‐mediated apoptotic pathway, and that the combination of Aβ and high Hcy activates this pathway in an additive manner. We showed that AβQ22 and Hcy additively increase DR4 and DR5 expression–known to follow these DRs activation (Micheau et al., 1999; Poh et al., 2007; Rossin et al., 2009)–, as well as promote the downregulation of cFLIP, the endogenous inhibitor of caspase 8, leading to an additive increase in caspase 8 activation. Activation of caspase 8 leads to both direct caspase 3 activation, as well as Bid cleavage, which results in increased mitochondrial membrane permeability (Kim et al., 2000), CytC release from the mitochondria, caspase 9 activation, and ultimately caspase 3 activation. Indeed, AβQ22 and Hcy also produce an additive increase in Bid cleavage, caspase 9 activity and a synergistic increase in caspase 3 activity.

Aβ peptides, including the E22Q variant, are known to promote mitochondria‐mediated apoptosis (Fossati et al., 2010; Solesio et al., 2018). Hcy was also found to promote mitochondria‐mediated apoptotic pathways within ECs, through a decrease in BCL2/Bax mRNA ratio and an increase in CytC release and caspase 9 activity (Tyagi et al., 2006). Here, we demonstrated that combined treatment of cerebral ECs with AβQ22 and Hcy results in potentiated decreases in BCL2 and increases in Bax protein expression, which is responsible for the formation of the mitochondrial permeability transition pore (MPTP) (Chen et al., 2015; Narita et al., 1998; Kowaltowski et al., 2000), increased mitochondrial fragmentation and CytC release. A potentiated CytC release was indeed confirmed by both immunocytochemistry and ELISA, eventually inducing additive increases in DNA fragmentation (apoptosis) and secondary necrosis. Overall, these results demonstrate that all the steps of the DR‐mediated apoptotic pathway, which directly triggers mitochondria‐mediated apoptosis, are exacerbated in a sequential and additive manner by the combined challenge with Aβ and Hcy. In addition, we revealed that silencing of DR4 and DR5 protects against AβQ22 and Hcy mediated apoptosis, providing direct evidence that AβQ22 and Hcy operate through the same DR‐mediated apoptotic pathway to exacerbate cerebral EC apoptosis and that this specific apoptotic pathway may be a potential target for future therapeutic strategies to tackle the cerebrovascular damage induced by both amyloidosis and Hhcy.

Additionally, Aβ peptides, including the AβQ22 variant, have been shown to decrease TEER, indicative of an increased BBB permeability, (Parodi‐Rullan et al., 2020). Hhcy can also increase BBB permeability (Beard Jr. et al., 2011) (Kamath et al., 2006). This study is the first to demonstrate that combined treatment of HCMECs with AβQ22 and Hcy potentiates barrier dysfunction, operating in an additive manner on the same molecular mediators of BBB damage. In particular, we showed that Hcy exacerbates the Aβ‐mediated decrease in claudin5 and increased phosphorylation of claudin5 and VE‐cadherin, previously shown to be associated with BBB permeability and retraction of ECs from each other (Hatanaka et al., 2011; Sidibe & Imhof, 2014; Yamamoto et al., 2008). Importantly, combined treatment of HCMECs with AβQ22 and Hcy potentiates an early release of IL2. This cytokine is known to lead to the phosphorylation of VE‐cadherin, revealing a specific molecular mechanism through which the combination of AβQ22 and Hcy potentiates BBB permeability (Wylezinski & Hawiger, 2016). Acute treatment with AβQ22 and Hcy also additively increased the expression of ICAM, an extravasation molecule that promotes EC recruitment of immune cells into the brain (Dietrich, 2002), as well as MMP2 activation, which results in digestion of the extracellular matrix (Weekman & Wilcock, 2016).

At later time points, we also observed an additive decrease in ZO1, a vital anchor protein within the BBB, responsible for linking cytoskeletal proteins, such as actin, in one cell, and TJ proteins, such as cadherins, occludins, and claudins, in another adjacent cells (Fanning et al., 1998; Liu et al., 2012; Tornavaca et al., 2015), as well as for actin filaments organization and polymerization. Interestingly, we also observed within our immunofluorescent ZO1 experiments, a drastic decrease in nuclear ZO1 expression in HCMECs treated with AβQ22 and Hcy, compared to control cells, which demonstrated strong nuclear ZO1 expression. This effect of AβQ22 and Hcy on HCMECs is worth noting, since it has been found that ZO1 localizes to the nucleus prior to TJ maturation and during cell‐to‐cell contact remodeling (Gottardi et al., 1996). These findings, which we also confirmed in Tg2576 animals exposed to a Hhcy‐inducing diet, explains the dramatic loss of EC barrier integrity and TEER associated with the combined treatment. Accordingly, in patients with comorbid AD/CAA and HHcy, a severe BBB permeability may be expected. This finding is of particular importance in view of the recently FDA approved Aβ‐targeted immunotherapies and their probability to induce ARIA (amyloid‐related imaging abnormalities), with cerebral edema or hemorrhage, an undesired effect causally mediated by BBB dysfunction. Our data suggests that patients presenting with both amyloidosis (AD/CAA) and Hhcy should be considered at higher risk for these complications, due to their already highly compromised BBB function, and physicians should be cautious in prescribing Aβ immunotherapies in patients with these comorbidities. These findings also highlight the need to continue the search for alternative therapies which could be affective also in patients with cerebrovascular comorbidities (Canepa et al., 2023), which are among the most frequent AD associated comorbidities (Carey & Fossati, 2023).

Angiogenesis is an important repair mechanism, particularly needed when the brain is damaged or hypoperfused, such as in neurodegenerative conditions like AD and CAA. Aβ peptides, including the AβQ22 variant, have been shown to reduce cerebral EC angiogenic capabilities (Parodi‐Rullan et al., 2020). Hcy is also known to inhibit angiogenesis (Zhang et al., 2012) (Nagai et al., 2001) in peripheral ECs. This study elucidates Hcy's effect on cerebral EC angiogenesis and demonstrates that combined treatment of cerebral ECs with AβQ22 and Hcy potentiates the decreases in angiogenesis progression scores. Furthermore, we revealed that pro‐inflammatory cytokines such as IL13, IL4, IL8, IL1β, and IL6, known to induce pro‐angiogenic pathways (Ma et al., 2021), are additively decreased as early as 3 h in EC challenged with both Aβ and Hcy. IL13 and IL4 are both potent inducers of angiogenesis, stimulating the formation of tubular vessels by human microvascular ECs in vitro. Specifically, IL13 has been found to promote VEGF‐A production (Fukushi et al., 2000) (Fukushi et al., 1998). IL8 has been shown to regulate angiogenesis by stimulating VEGF expression and autocrine VEGFR2 activation in ECs as well as enhance EC proliferation and survival (Martin et al., 2009; Li et al., 2003). IL‐1β potently stimulates angiogenesis in vitro within endothelial progenitor cells (Rosell et al., 2009). IL6, a pleiotropic cytokine with pro‐ and anti‐inflammatory functions, has been shown to inhibit angiogenesis in some molecular contexts, but also to stimulate vessel sprouting in ECs and stimulate VEGF production (Gopinathan et al., 2015) (Zegeye et al., 2020). The effect on decreasing these cytokines is therefore a possible upstream mechanism through which AβQ22 and Hcy additively decrease the activation of angiogenic pathways.

Additionally, this study has been the first to demonstrate that combined challenge with Aβ and Hcy additively diminishes cerebral endothelial barrier wound healing capabilities. More specifically, Aβ and Hcy decrease actin polymerization kinetics, a process important for barrier formation as well as wound healing. Additionally, acute exposure to Aβ and Hcy decreased phosphorylation of VEGFR2 at tryosine1175 (Y1175), which has been associated with EC proliferation and angiogenesis (Wang, Bove, et al., 2020), as well as additively reduced the expression of VEGF‐A and diminished the release of soluble VEGF‐A by HCMECs. Mechanistically, VEGFR2 endocytosis is an essential regulator of angiogenesis signaling. VEGF binding to the VEGFR2 on the EC plasma membrane results in receptor endocytosis into the slow trafficking pathway where the receptor will end up in early endosomes. Internalization within early endosomes promotes VEGFR2 phosphorylation and helps to propagate the downstream signaling and prevent degradation or constitutive recycling of the receptor (Lampugnani et al., 2006). The observed additive decrease in colocalization of the VEGFR2 within early endosomes suggests a significant reduction in VEGFR2 internalization and subsequent pro‐angiogenic signaling, and may additionally suggest a potentiated disruption in autophagic mechanisms by Aβ and Hcy, which will need to be further investigated.

Importantly, key mechanisms uncovered by our in vitro experiments were confirmed in vivo in prefrontal cortex samples of Tg2576 and WT mice subjected to Hhcy‐inducing diet. WT mice with Hhcy and Tg2576 mice demonstrated trends towards increased DR4 expression and decreased ZO1 and pVEGFR2 Y1175 expression. However, in Tg2576 mice with Hhcy, an additive and significant upregulation of DR4 expression, downregulation of ZO1 and reduction in pVEGFR2 Y1175 were observed. These results provide in vivo evidence of Hhcy's ability to potentiate Aβ‐induced DR‐mediated apoptosis, BBB dysfunction, and angiogenic downregulation by modulating the same molecular mechanisms revealed by our HCMECs results. In addition, this study revealed that Tg2576 AD mice and Tg2576 mice with Hhcy demonstrate a significant upregulation of VEGF‐A within the prefrontal cortex, a differing result from our in vitro experiments, which looked specifically at cerebral EC VEGF‐A expression. Interestingly, it has been demonstrated that AD patients also express higher levels of VEGF within certain cerebral tissue regions (Ali et al., 2022; Mahoney et al., 2021; Thomas et al., 2015), while cerebral capillary specific expression of VEGF is depleted within AD patients (Provias & Jeynes, 2014), which coincides with our in vitro and in vivo results. Additionally, cerebral VEGF overexpression within the AD brain has been linked to pericyte loss, capillary stalls, increases in BBB permeability, decreased CBF and increased AD pathology (Ali & Bracko, 2022; Mahoney et al., 2021), indicating a maladaptive overexpression. It is becoming more accepted that aberrant VEGF/VEGFR2 signaling plays a large role within promoting AD pathology and cognitive decline and this study has begun to demonstrate that this maladaptive VEGF/VEGFR2 signaling, typical in dementia, may be exacerbated within Tg2576 mice with Hhcy. These mice demonstrated significantly elevated prefrontal cortex VEGF‐A levels together with significantly decreased pVEGFR2 Y1175 expression, suggesting that although VEGF‐A levels are increased in the brain, this ligand is not binding to the VEGFR2 to promote its phosphorylation, which is necessary for angiogenesis to be carried out, demonstrating evidence of VEGFR2 signaling dysfunction, particularly in the Tg2576 mice with comorbid Hhcy.

This study is not free of limitations. Mimicking the chronic condition of Hhcy in vitro presents challenges. Individuals with Hhcy have Hcy levels from 15 μM to over 100 μM concentrations, circulating throughout their blood and organs for years. To mimic acutely, for short treatments in vitro, the effects of the chronic physiological exposure to Hhcy, most previous studies on ECs utilized concentrations of 1 mM or higher, necessarily higher than physiological levels, with 1 mM being the initial concentration that activated endothelial apoptosis in most reports (Chen et al., 2021; Fan et al., 2019; Jia et al., 2013; Nagai et al., 2001). Considering that our in vitro treatments span from a few hours to 2 days, we decided to also utilize for most experiments a 1 mM concentration, the lowest concentration shown to induce apoptosis in previous EC studies, for our HCMECs. However, the corroborating results of our mouse brain experiments, where chronic treatment with Hhcy is performed for months at non‐lethal concentrations, serve to confirm the physiological validity of our in vitro data.

Additionally, future studies will be needed to investigate Hhcy's ability to potentiate Aβ‐induced DR‐mediated apoptosis, BBB dysfunction, and angiogenesis deficits in AD/CAA animal models and in human AD/CAA brains longitudinally with disease progression, in different cerebral regions and within isolated cerebral vessels. Finally, other pathways, such as autophagy or ER stress, may also be involved in the combined effects of Aβ and Hcy, and future research endeavors will be necessary to tackle these additional mechanisms.

## 5 CONCLUSIONS

Hhcy is a potent CV risk factor increasing pathology and memory impairments in AD mouse models, which is also a suggested risk factor for cognitive impairment in humans (Carey & Fossati, 2023). Overall, this study demonstrates that the combined exposure of cerebral ECs to Hcy and a vasculotropic Aβ peptide additively promotes TRAIL DR‐mediated apoptosis, BBB dysfunction, decreases cerebral EC repair mechanisms and promotes angiogenic impairment. Importantly, Hcy and Aβ act on the same molecular mechanisms within each pathway. EC death and barrier dysfunction are known to lead to a leaky BBB, resulting in microhemorrhages, infiltration of peripheral immune cells, neuroinflammation as well as decreased cerebral microvessel density and hypoperfusion. We show that angiogenesis and cerebrovascular wound healing, vital compensatory mechanisms capable of regenerating and repairing the brain microvasculature, are also additively damaged by the two challenges. Hence, this work suggests that the comorbid presence of Hhcy in AD and CAA patients will potentiate the vicious cycle of Aβ‐induced endothelial and BBB damage and inability to repair this damage, resulting in hypoperfusion, neurovascular unit dysfunction and poor clearance of amyloid, therefore contributing to a vicious cycle of increased amyloid deposition and toxicity. Ultimately, this study identifies specific endothelial pathways that are modulated in an additive manner by Hcy and Aβ to produce AD‐related cerebral microvascular pathology, revealing novel molecular targets for AD and CAA therapy. This work also paves the way to establishing possible preventive or therapeutic strategies to reduce the risk of dementia in individuals with CV risk factors such as Hhcy.

## AUTHOR CONTRIBUTIONS

AC and SF designed the study. AC conducted most of the experiments in this manuscript and wrote the manuscript. SF provided oversight and scientific insight on experimental planning, assisted and mentored AC in writing, edited the manuscript and obtained funding for this project. RPR conducted the ECIS TEER experiment. RVT bred and maintained the mouse cohorts utilized in this study and assisted with imaging and quantifying immunocytochemistry experiments. EC assisted with running western blots with mice samples. All authors read and approved the final manuscript.

## FUNDING INFORMATION

This work was supported by NIH R01NS104127 and R01AG062572 grants, the Edward N. and Della L. Thome Memorial Foundation Awards Program in Alzheimer's Disease Drug Discovery Research, the Alzheimer's Association (AARG‐20‐685,663), the Pennsylvania Department of Heath Collaborative Research on Alzheimer's Disease (PA Cure) Grant, awarded to SF, and by the Karen Toffler Charitable Trust, and the Lemole Center for Integrated Lymphatics research.

## CONFLICT OF INTEREST STATEMENT

We declare no conflict of interest.

## Supporting information


Appendix S1.

## Data Availability

The data included in this article will be shared upon reasonable request to the corresponding author and will be made available after publication in data biorepositories.
