# Supplementary material for: Homocysteine potentiates amyloid β‐induced death receptor 4‐ and 5‐mediated cerebral endothelial cell apoptosis, blood brain barrier dysfunction and angiogenic impairment
Source: Aging Cell. 2024 Feb 15;23(5):e14106. doi: 10.1111/acel.14106 (PMC11113365; doi:10.1111/acel.14106)
Supplement: Supplementary file 1 — Appendix S1. [file ACEL-23-e14106-s001.pdf]

Supplementary Figure 1

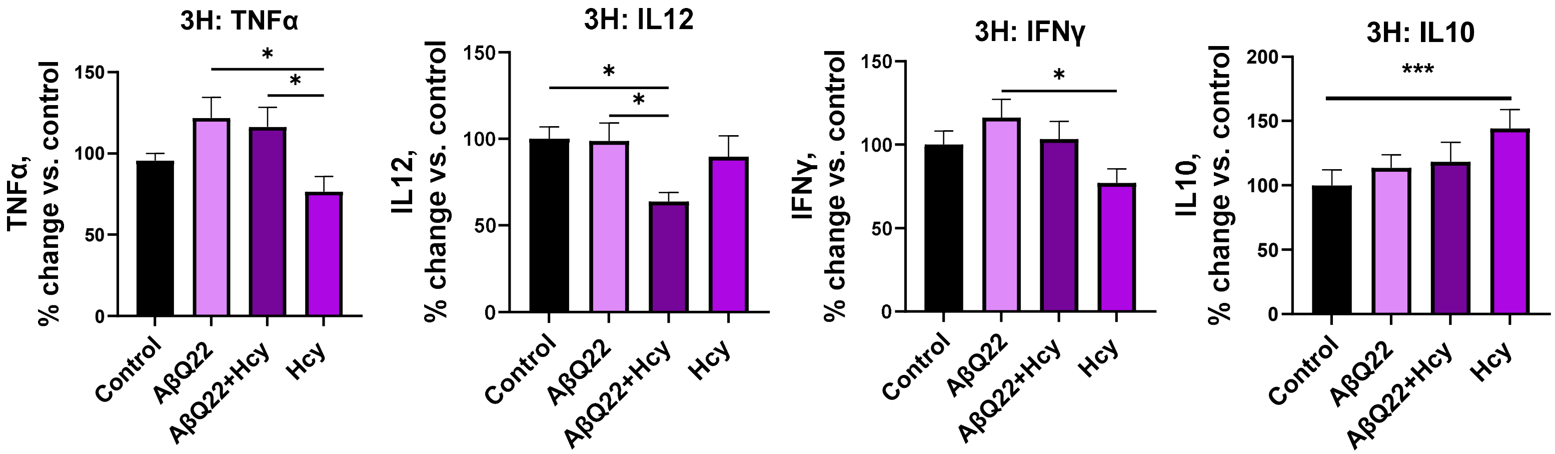

Supplementary Figure 1. Effects of Hcy and AβQ22 treatment on other pro-inflammatory cytokine release in HCMECs media. HCMECs were treated with 25uM AβQ22, 1mM Hcy, or a combination of the two for 3h and media was collected to run a multiplex inflammatory cytokine assay (MesoScale Discovery). Protein concentration was used for normalization. Data is represented as % change vs. control (N ≥ 3 experiments with 2 or more technical replicates; one-way ANOVA; Tukey's post-test). Hcy trended towards reduced TNFα release and significantly increased IL10 release compared to control cells. The combination of AβQ22+Hcy decreased the release of IL12 compared to control and AβQ22-treated cells.
